# Improving the solubility of pseudo-hydrophobic chemicals through co-crystal formulation

**DOI:** 10.1093/pnasnexus/pgaf007

**Published:** 2025-01-13

**Authors:** Isis Janilkarn-Urena, Amanda Tse, Jieye Lin, Bliss Tafolla-Aguirre, Alina Idrissova, Mindy Zhang, Samantha G Skinner, Nader Mostowfi, Jinah Kim, Nikhila Kalapatapu, Xinmin Chang, Christina Efthymiou, Christopher K Williams, Shino D Magaki, Harry V Vinters, Tamir Gonen, S Kaleem Ahmed, Hovhannes J Gukasyan, Daryl L Davies, Paul M Seidler

**Affiliations:** Department of Pharmacology and Pharmaceutical Sciences, University of Southern California Mann School of Pharmacy and Pharmaceutical Sciences, 1985 Zonal Ave, Los Angeles, CA 90089-9121, USA; Department of Pharmacology and Pharmaceutical Sciences, University of Southern California Mann School of Pharmacy and Pharmaceutical Sciences, 1985 Zonal Ave, Los Angeles, CA 90089-9121, USA; Department of Biological Chemistry, University of California, Los Angeles, 615 Charles E. Young Drive South, Los Angeles, CA 90095, USA; Howard Hughes Medical Institute, University of California, Los Angeles, Los Angeles, CA 90095, USA; Department of Neurology, David Geffen School of Medicine at University of California, Los Angeles, CA 90095, USA; Department of Pharmacology and Pharmaceutical Sciences, University of Southern California Mann School of Pharmacy and Pharmaceutical Sciences, 1985 Zonal Ave, Los Angeles, CA 90089-9121, USA; Department of Pharmacology and Pharmaceutical Sciences, University of Southern California Mann School of Pharmacy and Pharmaceutical Sciences, 1985 Zonal Ave, Los Angeles, CA 90089-9121, USA; Department of Pharmacology and Pharmaceutical Sciences, University of Southern California Mann School of Pharmacy and Pharmaceutical Sciences, 1985 Zonal Ave, Los Angeles, CA 90089-9121, USA; Titus Family Department of Clinical Pharmacy, University of Southern California Mann School of Pharmacy, Los Angeles, CA 90089, USA; Department of Pharmacology and Pharmaceutical Sciences, University of Southern California Mann School of Pharmacy and Pharmaceutical Sciences, 1985 Zonal Ave, Los Angeles, CA 90089-9121, USA; Department of Pharmacology and Pharmaceutical Sciences, University of Southern California Mann School of Pharmacy and Pharmaceutical Sciences, 1985 Zonal Ave, Los Angeles, CA 90089-9121, USA; Titus Family Department of Clinical Pharmacy, University of Southern California Mann School of Pharmacy, Los Angeles, CA 90089, USA; Department of Pharmacology and Pharmaceutical Sciences, University of Southern California Mann School of Pharmacy and Pharmaceutical Sciences, 1985 Zonal Ave, Los Angeles, CA 90089-9121, USA; Department of Pharmacology and Pharmaceutical Sciences, University of Southern California Mann School of Pharmacy and Pharmaceutical Sciences, 1985 Zonal Ave, Los Angeles, CA 90089-9121, USA; Department of Pathology and Laboratory Medicine, David Geffen School of Medicine at University of California, Los Angeles, CA 90095, USA; Department of Pathology and Laboratory Medicine, David Geffen School of Medicine at University of California, Los Angeles, CA 90095, USA; Department of Pathology and Laboratory Medicine, David Geffen School of Medicine at University of California, Los Angeles, CA 90095, USA; Department of Neurology, David Geffen School of Medicine at University of California, Los Angeles, CA 90095, USA; Department of Biological Chemistry, University of California, Los Angeles, 615 Charles E. Young Drive South, Los Angeles, CA 90095, USA; Howard Hughes Medical Institute, University of California, Los Angeles, Los Angeles, CA 90095, USA; Department of Physiology, University of California, Los Angeles, 615 Charles E. Young Drive South, Los Angeles, CA 90095, USA; University of Southern California Medicinal Chemistry Core Laboratory at the Alfred E. Mann School of Pharmacy and Pharmaceutical Sciences, Los Angeles, CA 90095, USA; Department of Pharmacology and Pharmaceutical Sciences, University of Southern California Mann School of Pharmacy and Pharmaceutical Sciences, 1985 Zonal Ave, Los Angeles, CA 90089-9121, USA; Titus Family Department of Clinical Pharmacy, University of Southern California Mann School of Pharmacy, Los Angeles, CA 90089, USA; Department of Pharmacology and Pharmaceutical Sciences, University of Southern California Mann School of Pharmacy and Pharmaceutical Sciences, 1985 Zonal Ave, Los Angeles, CA 90089-9121, USA

## Abstract

Natural products are ligands and in vitro inhibitors of Alzheimer’s disease (AD) tau. Dihydromyricetin (DHM) bears chemical similarity to known natural product tau inhibitors. Despite having signature polyphenolic character, DHM is ostensibly hydrophobic owing to intermolecular hydrogen bonds that shield hydrophilic phenols. Our research shows DHM becomes ionized at near-neutral pH, allowing the formulation of salts with transformed solubility. The MicroED co-crystal structure with trolamine reveals DHM salts as metastable co-crystalline solids with unlocked hydrogen bonding and a thermodynamic bent to solubilize in water. All co-crystal formulations show better inhibitory activity against AD tau than the nonsalt form, with efficacies correlating to enhanced solubilities. In vitro and in vivo pharmacokinetic measures demonstrate that DHM co-crystals display enhanced absorption and distribution with altered rates of elimination, suggesting that co-crystal formulations could be strategically used to fine-tune delivery properties. These results underscore the role of structural chemistry in guiding the selection of solubilizing agents for chemical formulation. We propose DHM co-crystals are appropriate formulations for research as dietary supplements to promote healthy aging by combating protein misfolding, although central nervous system (CNS) delivery remains a major limitation. DHM may be a suitable backbone for medicinal chemistry and possible development of pharmaceuticals with enhanced CNS exposure.

**Significance Statement**

There is a pressing need to identify chemicals that are suitable for promoting healthy aging. Our research introduces a valuable concept with the innovation of dihydromyricetin (DHM) co-crystal formulations that display improved PK/PD properties. DHM behaves as a hydrophobic substance despite having numerous hydrophilic substituents that conventionally drive water solubility. Crystalline solids are poorly water soluble due to a large solvation energy. Co-crystal formulations alleviate the thermodynamic barrier to solvation by disrupting intermolecular crystal contacts, thereby increasing the solubility and bioactivity of DHM co-crystals. These studies open the door for improved oral delivery of DHM by crystal engineering.

## Introduction

Solubility is a crucial factor affecting the efficacy of pharmaceuticals and other bioactive compounds, such as vitamins, natural products, and dietary supplements. The degree of absorption is directly proportional to the solubility of a compound, although the physical and chemical mechanisms underlying solubility are largely theoretical. Compounds that deviate from ideality pose a challenge to scientists seeking to troubleshoot and develop rational strategies to overcome poor solubility. Approximately, 40% of new pharmaceutical chemicals developed by the industry are described as practically insoluble in water (1), and nearly 90% of new drugs are classified accordingly as class II/IV in the Biopharmaceutics Classification System (2) partly due to low solubility. Crystal and salt formulations are a common strategy for preparing stable, safe, and bioavailable dosage forms in the pharmaceutical industry (3). However, predicting optimal co-crystal forms and how they will function remains challenging.

Like salts, co-crystal solid forms allow for higher dissolved concentrations in bulk solution. The present work investigates the solubility, activity, and microcrystal electron diffraction (MicroED) (4, 5) structure of co-crystal formulations of the natural product dihydromyricetin (DHM), a brain-permeable flavonoid with high chemical similarity to a known in vitro tau inhibitor, epigallocatechin gallate (EGCG), which is involved in Alzheimer’s disease (AD) pathology (6). DHM is also an antioxidant (7, 8) and anti-inflammatory (9–11) with potential benefits in ameliorating dyslipidemia (8, 9, 12, 13) and alcohol intoxication (14, 15).

Despite having rich phenolic character, DHM behaves as a hydrophobic substance with an aqueous solubility of only ∼0.4 mg/mL. While the hydrophobic character contributes to cell membrane permeation, poor water solubility impedes oral dosing and intestinal absorption. Water-soluble co-crystalline formulations of DHM could overcome dosing issues by enabling delivery of higher concentrations to increase absorption and distribution. While natural flavonoids and DHM are generally regarded as safe (GRAS), the feasibility of isolating them as salts or co-crystals with suitable counterions that improve poor physicochemical properties (i.e. low solubility, instability, or hygroscopicity) has not been widely explored (16).

Here, we investigate co-crystal formulations of DHM with counterions from the FDA-approved Inactive Ingredients Database (17) as inhibitors of AD tau tangles. With the aging population expected to double by 2050, and the US Department of Health and Human Services aiming to prevent and treat AD by 2025 (18), there is a pressing need for public health action. Epidemiological research suggests that lifestyle and environmental factors, along with genetics and aging, influence the development of AD. Current strategies to enhance healthy aging include attention to diet, exercise, and cardiovascular health. However, the lack of biologically active chemicals, such as pharmaceuticals and dietary supplements, impairs efforts to support healthy aging through early chemical intervention. Tau tangles are implicated in neuronal death in AD and other tauopathies, making the suppression of tau aggregation and spreading a top therapeutic interest. The structural similarity of DHM to EGCG prompted us to investigate DHM co-crystals as potential tau inhibitors.

## Results

### Effects of DHM and co-crystals on prionogenic seeding by AD tau

**Fig. 1.**
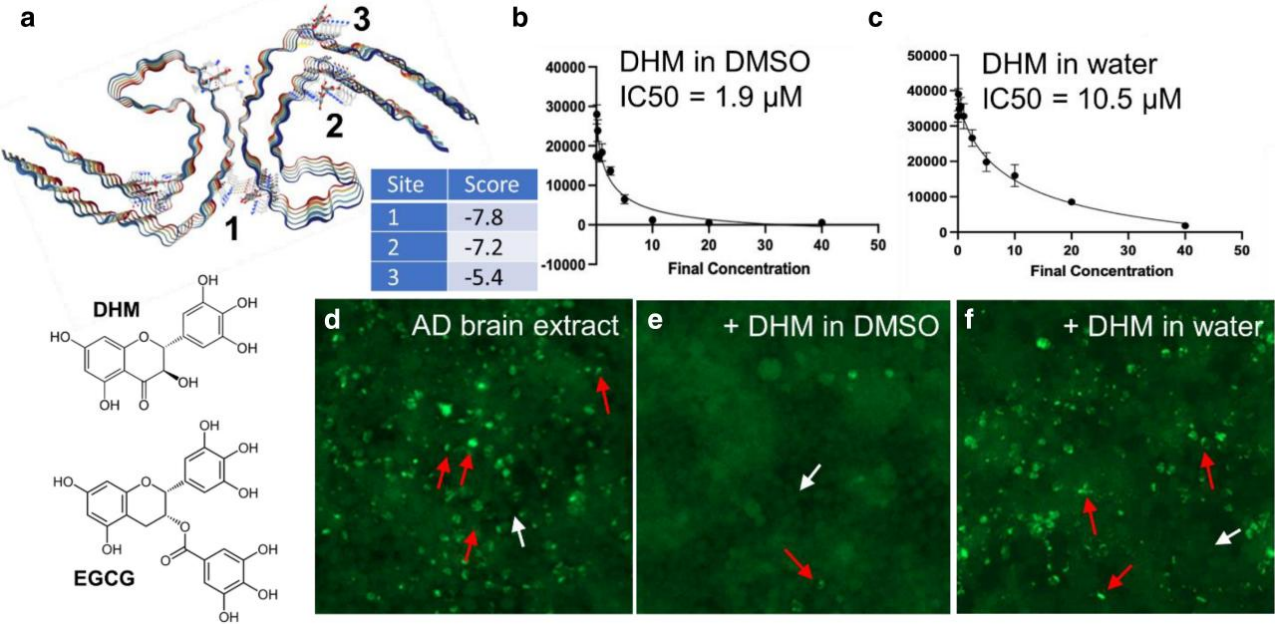
a) DHM-binding sites on AD tau fibrils predicted by CB-Dock. AutoDock Vina docking scores are shown in the embedded table. Chemical structures comparing DHM and EGCG are shown below the CB-Dock model. Chemical structure images from the public domain were obainted from Wikipedia. b and c) Dose-dependent seeding inhibition was measured by transfecting AD crude brain homogenates in HEK293 tau biosensor cells that stably express P301S 4R1N tau fused to YFP. Seeding inhibition was determined by counting the number of fluorescent puncta as a function of inhibitor concentration. IC_50_ values were calculated by nonlinear curve fitting from dose–response plots. Experiments in b were performed by preincubating AD crude brain homogenates with DHM dissolved in DMSO. Experiments in c were performed identically except using the soluble fraction of DHM dissolved in water following centrifugation at 8,000 rpm. d–f) Representative images from tau biosensor cells transfected with AD crude brain homogenate (d), or identically treated cells following preincubation of AD crude brain homogenate with DHM (10 mM final concentration on cells). Representative tau-4R1N cells containing aggregated tau puncta are marked with red arrows, and cells without are marked with white arrows.

The polyphenol DHM has a similar chemical structure to EGCG, a known polyphenolic ligand and inhibitor of tau (Fig. 1a). Therefore, we evaluated the in silico predicted binding of DHM to AD tau paired helical filaments (PHFs) using CB-Dock (19), a suite that incorporates cavity detection with AutoDock Vina. Using an AD tau PHF co-cryoEM structure with EGCG as a starting model, we evaluated the most likely binding cavities for DHM. The top-scoring predicted DHM-binding cavity matched the primary EGCG binding volume formerly determined by CryoEM (Fig. 1a), although the DHM-binding pose output by CB-Dock differs from models we generated manually by superimposing DHM with EGCG from the liganded CryoEM structure (Fig. S1). Both modeling approaches predict interactions with Lys340, although models generated by CB-Dock orient the *π* orbitals of aromatic moieties of DHM perpendicular to the fibril axis, whereas manually generated models orient stacks of aromatic moieties parallel to strand-forming *β* sheets of the fibril. Additional possible DHM-binding sites detected by CB-Dock include cavities labeled sites 2 and 3 (Fig. 1a). However, both Sites 2 and 3 scored lower by AutoDock Vina.

We tested the in vitro activity of DHM to inhibit prionogenic seeding by AD tau (Fig. 1b–f) since modeling suggests DHM can bind to AD tau fibrils in a manner similar to EGCG. Transfecting AD crude brain homogenates in tau biosensor cells, which express an aggregation-prone fragment of tau fused with YFP reporter, produces a punctated cellular phenotype, shown in Fig. 1d that results from the aggregation of intracellular tau that is seeded by tau from AD brain homogenate. Pretreating AD crude brain homogenates with DHM dissolved in DMSO inhibited seeding in a dose-dependent manner with an IC_50_ of 1.9 µM (Fig. 1b). In comparison, DHM that was dissolved in water elicited less potent inhibition with an apparent IC_50_ of 10.5 µM (Fig. 1c). These results were consistent with limiting solubilities observed for DHM stock solutions prepared in water (Fig. S2). We observed that 10 mM master stock solutions of DHM prepared in water were unstable, resulting in DHM precipitation and eventual equilibration to a stock solution with actual measured concentrations of 0.65 mg/mL (2 mM). These results are consistent with the reported solubility for DHM of ∼0.2–0.4 mg/mL, which is only slightly soluble in water despite rich phenolic character. In contrast, stock solutions prepared in DMSO remained soluble. Our observation that only about 20% of DHM remained dissolved in aqueous solutions after clearing insoluble matter by centrifugation could account for the lowered measured IC_50_ of DHM solutions that were prepared in water. These data illustrate two important points: (ⅰ) DHM effectively inhibits seeding by AD tau, particularly when solubilized in polar, aprotic solvents, and (ⅱ) the inhibitory power of DHM is limited by poor aqueous solubility.

Compounds with limited aqueous solubilities exhibit low bioavailability and compromised bioactivity. Therefore, we investigated the physicochemical factors underlying the poor aqueous solubility of DHM with the goal of developing salt formulations to overcome the solubility limitation. Analysis of pK_a_, shown in Fig. S3, reveals plausible ionization states for DHM near-neutral pH, and the experimental pK_a_ was verified to be pH 7.9. Therefore, we investigated the solubility-enhancing properties of the crystals/salts formed. Counterion screening with pharmaceutically relevant counterions: triethanolamine (TEA), sodium hydroxide (NaOH), TRIS Base (2-amino-2-(hydroxymethyl)-1,3-propanediol), L-lysine, and calcium hydroxide (Ca(OH)_2_), yielded powders taken as potential co-crystal formulations. As detailed in the Supplementary Text and Table S2, these included DHM-TEA prepared from ethanol, DHM-Tris prepared from isopropanol, DHM-Lysine prepared from water, and two DHM-Ca formulations: DHM-Ca(b) and DHM-Ca(c), prepared from methanol and 2-propanol, respectively.

**Fig. 2.**
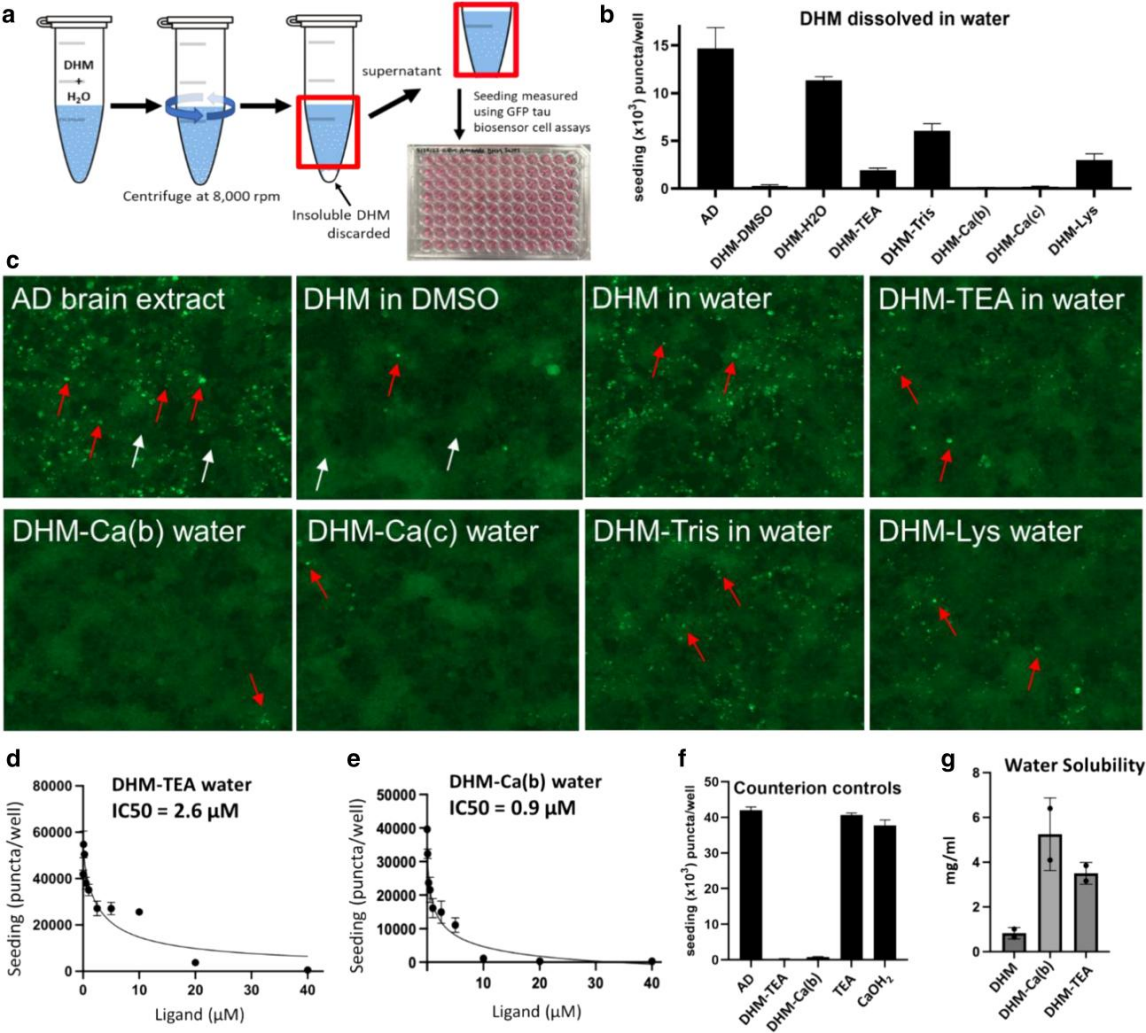
a) Schematic showing experimental design. Co-crystalline DHM formulations were dissolved in water to a target 10 mM concentration. Insoluble matter was removed by centrifugation as indicated. Supernatants containing water-solubilized DHM were assayed for tau inhibitor activity. b) Seeding inhibition measured by quantifying the number of fluorescent puncta as a function of the indicated inhibitor. AD sample is AD brain homogenate without added inhibitor. Error bars represent SDs of triplicate measures. c) Representative images from b of tau biosensor cells seeded by transfection by AD crude brain homogenates following pretreatment with DHM or salt co-crystals. Inhibitors were added to a final concentration of 10 µM on cells. Example puncta are shown by red arrows, examples of nonseeded cells are shown with white arrows. d and e) IC_50_ dose-dependent inhibition of puncta following pretreatment with DHM-TEA and DHM-Ca(b). f) Seeding inhibition measured for DHM-TEA and DHM-Ca(b), or TEA and DHM-Ca(OH)_2_. g) Solubility determination of DHM, DHM-TEA, and DHM-Ca(b) in water, expressed in mg/mL.

Tau inhibitor activity for the five obtained DHM co-crystals with counterions was prepared by dissolving in water to achieve a target concentration of 10 mM. Our experimental workflow, depicted in Fig. 2a, involved clearing insoluble DHM from concentrated stock solutions through centrifugation, resulting in supernatants that were close to the saturation limit. These supernatants were used to prepare DHM dilutions for tau inhibitor testing. We reasoned that the aqueously soluble fraction of DHM co-crystals would exhibit tau inhibitor activities approaching those of DHM dissolved in DMSO since more DHM is expected to dissolve in the aqueous phase from co-crystal formulations. All the co-crystal forms tested demonstrated greater tau inhibitor activity compared with DHM alone, as shown in Fig. 2b–e. DHM-TEA and DHM-Ca(b) exhibited the most potent improvements in tau inhibition, with IC_50_ values of 2.6 and 0.87 µM, respectively (Fig. 2d and e), similar to the IC_50_ observed for DHM dissolved in DMSO.

To ensure counterions themselves do not contribute to inhibition of tau seeding, we measured seeding by preincubating AD brain homogenates with counterions. As shown in Fig. 2f, neither TEA nor Ca(OH)_2_ exhibited inhibitory activity toward tau seeding by crude AD brain homogenates. This finding confirms that the inhibitor activity of DHM co-crystals is due to the enhanced solubility of DHM and is not the result of any synergistic inhibition by the accompanying counterions. Also supporting these data are Fig. S5 showing that DHM co-crystals, DHM-TEA and DHM-Ca(b), dissolved in DMSO, rather than water, demonstrated IC_50_ values of ∼1 µM, which is comparable with the IC_50_ of DHM dissolved in DMSO. These data indicate that the counterions, themselves, in the co-crystal formulations do not augment the tau inhibitor activity beyond what is achieved by DHM itself. Thus, the mechanism by which counterions enhance the inhibitory activity of DHM is by improving DHM’s solubility. The enhancement in solubility is corroborated by experimental solubility data for DHM-TEA and DHM-Ca, as shown in Fig. 2g, which reveals a 4- to 6-fold increase in water solubility, consistent with the fold improvements observed in IC_50_ values for these co-crystals.

Since DHM shares a similar structure to EGCG, we tested the hypothesis that DHM inhibits seeding by a similar mechanism as AD tau disaggregating fibrils. AD tau fibril disaggregation by DHM was measured by quantitative electron microscopy. As shown in micrographs in Fig. S6, incubation with EGCG reduces the average number of AD tau fibrils observed by negative-stain EM imaging by 70–80%. DHM-TEA and DHM-Ca(b) both reduced fibril density by 50%, from 300 to 160 fibrils after 48 h incubation with DHM-TEA and from 227 total fibrils to 111 fibrils after 48 h incubation with DHM-Ca(b). These data suggest fibril disaggregation is one plausible mechanism of inhibition, although they leave open the possibility that DHM binding to AD tau is inhibitory toward tau seeding since fibril disaggregation occurs to a lesser extent compared with EGCG. Further supporting the possibility that ligand binding, itself, exerts inhibitory effects absent of fibril disaggregation are data showing that inhibitor preincubation is not needed to inhibit seeding (Fig. S7). These data show that seeding inhibition by DHM occurs despite some fraction of remaining fibrils in solution, suggesting it is possible that ligand binding itself suppresses seeding, at least to a partial degree.

### Structural chemistry of DHM co-crystals

**Fig. 3.**
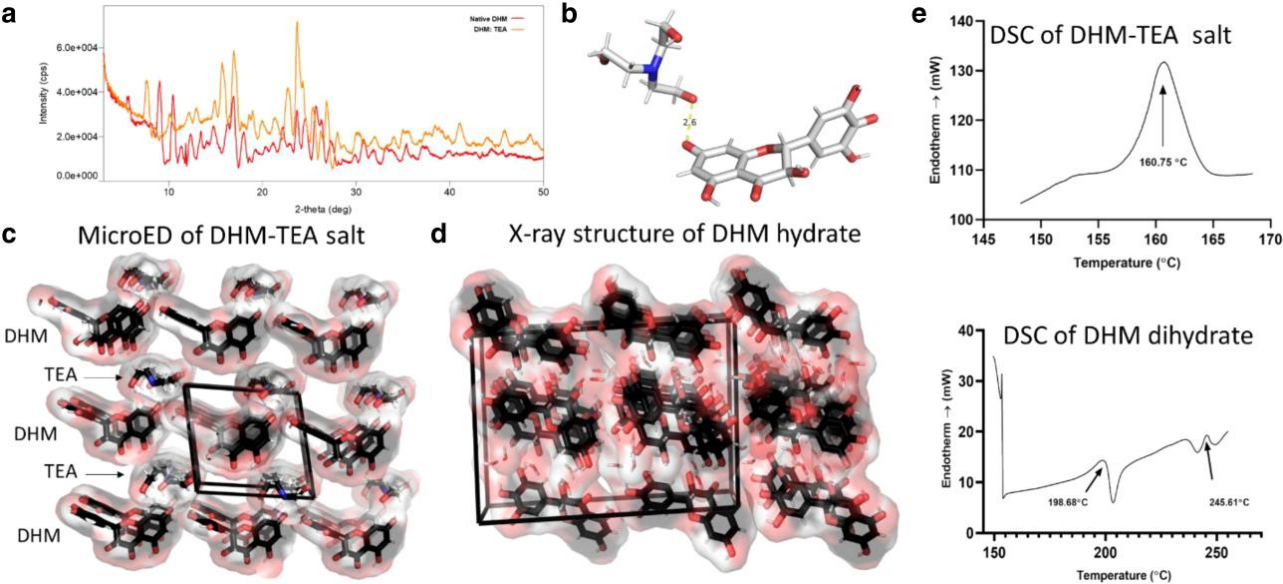
a) X-ray powder diffractogram comparing native DHM shown in red, overlaid against co-former DHM with TEA shown in orange. b) Asymmetric unit of DHM complex with TEA determined by MicroED. c) Crystal lattice of DHM-TEA salt. Note solvent channels created by the intercalation of TEA in the lattice between DHM molecules. d) X-ray structure of DHM dihydrate (20). Differential scanning calorimetry thermographs of DHM-TEA and DHM dihydrate.

The mechanism of enhanced solubility for DHM salts was investigated by structural chemistry. DHM-TEA and DHM-Ca exhibited X-ray powder diffraction indicating crystallinity (Figs. 3a, S8, and S9), although only DHM-TEA produced suitable diffraction by MicroED to yield an atomic structure. TEA is seen in the 0.74 Å resolution MicroED structure in a 1:1 molar ratio positioned 2.6 Å from the O_2_ of the phenol of the resorcinol that is predicted to first become ionized (Figs. 3b and S3a). Crystals of DHM-Ca(b) also diffracted, but diffraction power was insufficient to enable atomic structure determination. It is possible the tridentate nature of TEA reinforced the crystal lattice to enable stronger diffraction and structure elucidation compared with the DHM-Ca salts, which were crystalline but exhibited weaker diffraction.

DHM-TEA co-crystals have large solvent channels that enable solubilization. Comparing DHM-TEA with the formerly published crystal structure of DHM dihydrate (20) (Fig. 3c and d) reveals large solvent channels and high solvent content in the DHM-TEA lattice. The DHM-TEA crystal lattice is more porous, with an increased void volume of 83.3% and a Matthew coefficient (*V*_M_) of 2.07 Å^3^/Da (assuming a mass of 469 Da and a unit cell volume of 971.5 Å^3^), which is atypically large for a small molecule crystal. The large *V*_M_ reflects high solvent content in the DHM-TEA lattice and more closely resembles a protein crystal that is entrenched with large solvent channels and high solvent content (21). An analysis of porosity using CrystalMaker reveals the DHM dihydrate unit cell has 79.4% void space and a unit cell volume of DHM dihydrate, 2,899.1 Å^3^. The *V*_M_ of DHM dihydrate, 1.02 Å^3^/Da, is comparable with other small molecule crystals with low solvent content. Correspondingly, DHM-TEA co-crystals exhibit a cooperative thermodynamic transition seen in DSC melt curves at a lower temperature of 160 °C, compared with the shallow biphasic transition that is seen for DHM dihydrate (Fig. 3e). These data indicate that the DHM-TEA formulation is less thermodynamically stable than DHM dihydrate, which is consistent with structural and macroscopic measures showing that DHM co-crystal possesses higher solvent content and is more readily dissolved in water due to the presence of water channels that are absent from the dihydrate form.

**Fig. 4.**
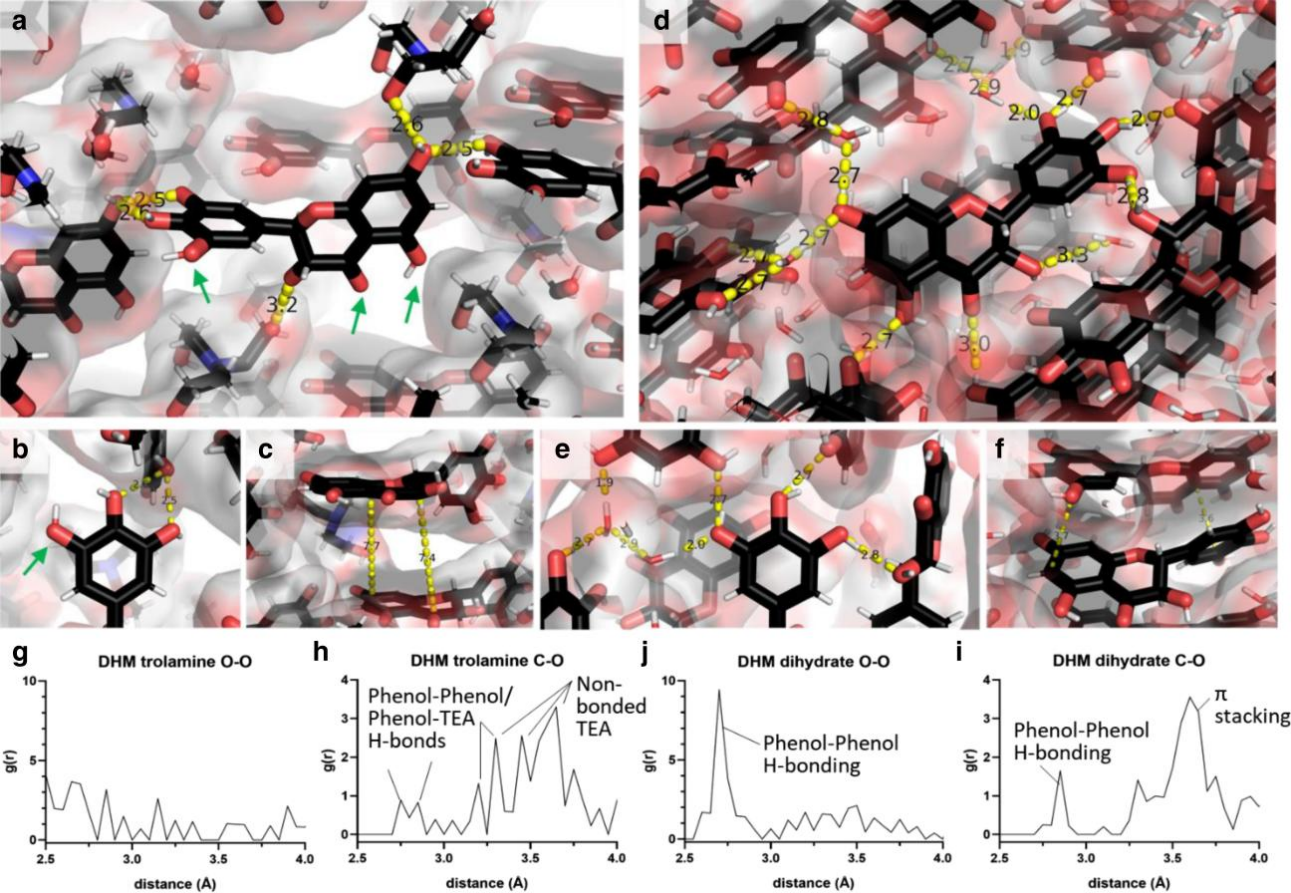
a–c) DHM-TEA MicroED structure. Nonbonded groups are shown in A and B with green arrows. Contacts between phenols of the pyrogallol ring are shown in (b), and aromatic stacking interactions are shown in (c) for DHM-TEA. (d–f) As in a–c, except shown for the DHM dihydrate X-ray structure (20) for comparison. (g and h) Pair correlation functions for DHM-TEA and DHM dihydrate, as labeled.

TEA disrupts intermolecular H-bonding networks that shield phenols from water, thereby increasing solubility. As shown in Fig. 4a and b, TEA molecules disrupt H-bonding between phenols of the pyrogallol and resorcinol rings. Otherwise, all the phenols of DHM are engaged in H-bonded interactions, as shown in the dihydrate crystal (Fig. 4c and d). Importantly, trolamine occupies spaces in the lattice without contacting every phenol. Three potential H-bonding groups of DHM are left unpaired in the DHM-TEA lattice. The angles at which trolamines intercalate relative to the pyrogallol ring, ∼145–155°, leave gaps of ∼3.5 Å, which is sufficient in space and geometry to allow water molecules to infuse into the lattice of the co-crystal and hydrate the nonpaired H-bonding groups.

The improved solvation properties of DHM-TEA can be attributed to fewer intermolecular hydrogen bonds (three, as shown in Fig. 4a and b compared with nine, as depicted in Fig. 4d and e for DHM dihydrate). Differences in the H-bonding network of the two lattices are evident when examining the pair correlation functions, *g*(*r*), shown in Fig. 4g–j. In the DHM dihydrate crystal lattice (Fig. 4j), a single peak at 2.7 Å dominates the O–O *g*(*r*), reflecting an extensive regular network of intermolecularly H-bonded phenols that anneal DHM molecules in the dihydrate lattice (shown in Fig. 4d and e). Similarly, a peak at 2.85 Å is observed in the C–O pair correlation function in Fig. 4i, arising from intermolecularly H-bonded phenols. Conversely, the phenols in the DHM-TEA lattice exhibit fewer H-bonds, as evident by the absence of peaks in the O–O pair correlation function (Fig. 4g). Minor peaks are discernible in the C–O function at 2.75 and 2.85 Å (Fig. 4h), corresponding to two phenol–phenol interactions and an H-bonded TEA, as shown in Fig. 4a and b. Additionally, a second peak in the DHM-TEA C–O pair correlation function in Fig. 4h occurs at ∼3.2 Å, corresponding to the H-bonded distance between a phenol and TEA molecule.

The lower frequency of contacts in the DHM-TEA crystal lattice agrees with its lower transition temperature, as measured by DSC for DHM-TEA (Fig. 3e) and explains the structural mechanism of increased solubility. A lower solvation energy for DHM-TEA arises from enthalpic contributions since nonbonded phenols (Fig. 4a, green arrows) are free to H-bond to solvating water molecules. In contrast, solvation of DHM dihydrate necessitates the exchange of intermolecular lattice H-bonds for water molecules, a process that is both kinetically and thermodynamically unfavorable given the absence of a net enthalpic gain to offset the entropic solvation penalty.

Hydrophobic contacts seen in the crystal further lower the energy of solvation. The C–O *g*(*r*) function shows a peak at 3.7 Å, corresponding to the *π* stacking distances of aromatic rings of DHM in the dihydrate lattice (Fig. 4f and i). The ring-to-ring distance increases to 7.5 Å in the DHM-TEA lattice (Fig. 4h and e) indicating loss of *π* stacking, although a peak in the C–O *g*(*r*) is seen between 3.4 and 3.8 Å, which arises from nonbonded distances between TEA molecules in the crystal lattice. Loss of *π* stacking from the DHM-TEA lattice is expected to lower further the entropic barrier to solvation since aromatic rings of DHM in the TEA salt can be partially solvated in crystallo. Thus, the configuration in TEA in the co-crystal lattice eliminates the energetic barrier that is associated with breaking *π*-stacking interactions to solubilize DHM.

### In vitro absorption, distribution, metabolism, and excretion (ADME) characteristics for DHM co-crystals

**Fig. 5.**
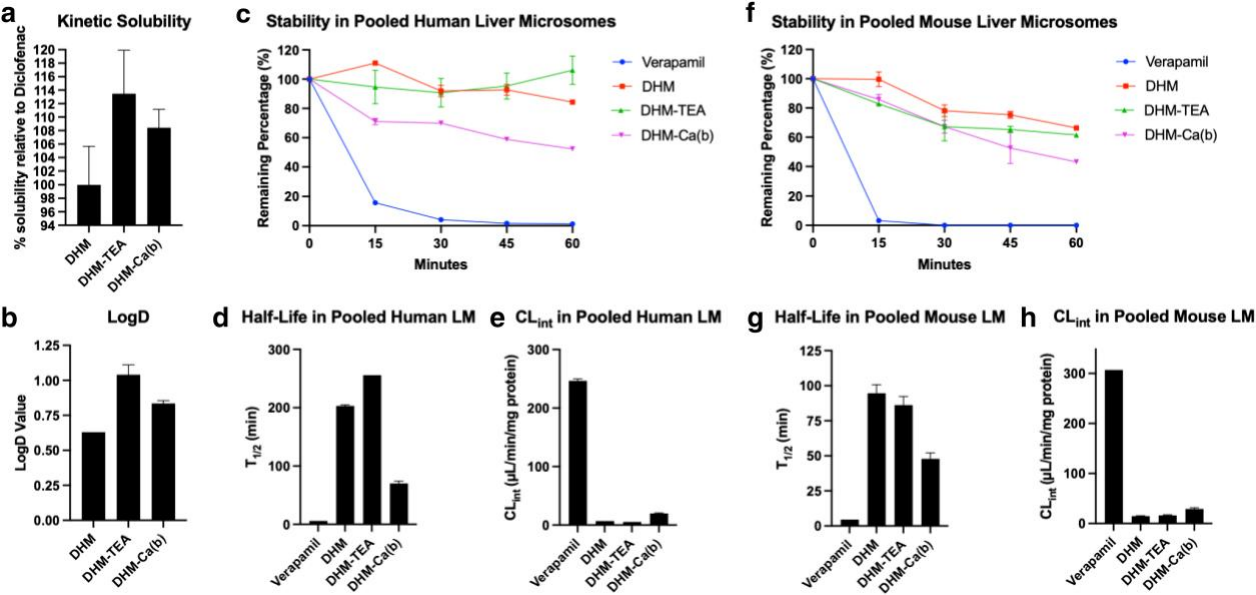
In vitro PK data. a) Kinetic solubility of DHM, DHM-TEA, and DHM-Ca(b) measured in percent solubility relative to Diclofenac. b) LogD values of DHM, DHM-TEA, and DHM-Ca(b). (c–h) Metabolic stability of Verapamil, DHM, DHM-TEA, and DHM-Ca(b) in pooled human LM (c–e) and in pooled male mouse LM (f–h). Error bars represent the SD of duplicate measures.

To obtain insight into the pharmacokinetic (PK) characteristics of DHM and the effects of co-crystal formulation, we investigated the in vitro ADME properties that are shown in Fig. 5. Kinetic solubility in simulated intestinal fluids (SIFs) is a key indicator of the equilibrium dissolution and absorption. In the context of oral drugs and supplements, poor aqueous solubility can hinder intestinal absorption. Consequently, we examined the solubility of DHM and its co-crystal forms in relation to diclofenac, a nonsteroidal anti-inflammatory drug with poor aqueous solubility. As illustrated in Fig. 5a, DHM demonstrated a solubility profile similar to that of diclofenac, which exhibits a kinetic solubility of ∼300 µM in SIF. DHM-TEA and DHM-Ca(b) exhibited kinetic solubilities in SIF that were 10–20% higher.

The distribution of DHM to the hydrophobic phase, LogD, reflects lipophilicity and the potential cell membrane permeability. Central nervous system (CNS) drugs have an average LogD of 1.7 with a peak in the Boltzmann distribution between 1 and 3 (22). LogD values for DHM-TEA and DHM-Ca(b) increased from 0.63 for DHM to 1.04 and 0.84, respectively (Fig. 5b). The LogD signifies overall improved permeability for co-crystal formulations, possibly due to enhanced solubilization and corresponding increases in solute concentrations.

Metabolic stability assays conducted using pooled human liver microsomes (LMs) in comparison with the control drug, verapamil, reveal remarkable stability for DHM and the DHM-TEA co-crystal, with nearly 100% of DHM remaining after a 60-min incubation period (Fig. 5c). DHM’s stability in LM, as demonstrated in Fig. 5c–e, is greater in human LM than in mice, as shown in Fig. 5f–h. In both cases, DHM exhibits superior stability when compared with verapamil, which is primarily metabolized by liver enzymes. In human LM, the half-life *T*_1/2_ for DHM is estimated to be 203 min, contrasting with the comparatively shorter *T*_1/2_ of 94 min in mouse LM. DHM-1a exhibits the greatest stability in human LM, but *T*_1/2_ is similar to DHM in mouse LM. Conversely, DHM-Ca(b) demonstrates moderate elimination kinetics in human LMs with a *T*_1/2_ of 70 min accompanied by an increased rate of intrinsic clearance (CL_int_) of 20 µL/min/mg protein (Fig. 5e and h).

### Serum and brain exposure for DHM co-crystal formulations

**Fig. 6.**
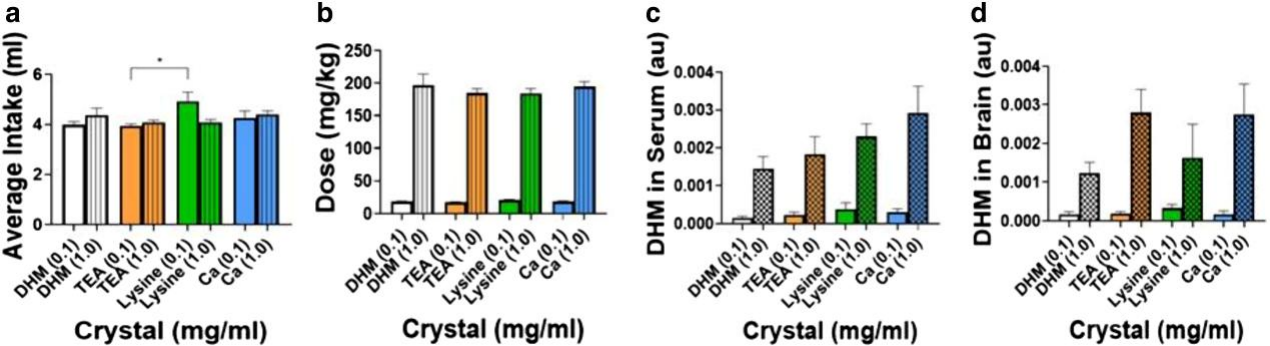
In vivo male and female c57BL/6 mice (*n* = 4/sex/group) PK data showing: a) average fluid intake, free DHM, DHM-TEA, DHM-Lysine, and DHM-Ca(b) crystals dissolved into the drinking water and administered orally for 10 days (*0.04), b) daily average dose for each group, c) terminal DHM exposure quantified from serum, and d) terminal DHM exposure quantified from whole brain homogenates.

The effective delivery of DHM by an oral route is an important requisite for its use as a dietary supplement. To evaluate improved oral delivery of DHM co-crystals in animals, we dissolved DHM in drinking water and assessed steady-state levels in serum and brain. Orally administered DHM was delivered by dissolving DHM co-crystal powders in drinking water that mice had ad libitum access to throughout the 10-day study duration. The solutions were well tolerated by the mice independent of dose, and mice showed no significant or concerning impacts on the daily fluctuations in body weight or food intake (Fig. S11a and b). Overall, as demonstrated in Fig. 6a, mice from all groups consumed an average of 4.2 mL/day showing no profound preference or aversion for any of the DHM formulations. Notably in the low-dose DHM + lysine group, mice drank more, averaging 4.9 mL/day compared with the DHM + TEA group, at the same dose (Fig. 6a; *0.04). Total DHM exposures, summarized in Fig. 6b, amount to an average of 18.6 mg/kg in the low-dose groups and 177 mg/kg in the high-dose groups, or an average daily dose of 0.43 mg for the low-dose groups and 4.2 mg for the high-dose groups (Fig. S11c).

Serum and brain levels following 10-day oral exposure were evaluated to ascertain steady-state concentrations (*C*_ss_) for DHM. Liquid chromatography-mass spectrometry (LCMS) data in Fig. 6c and d show terminal levels of DHM for each group on the 10th day of continuous oral administration. Serum levels trended higher, indicating improved absorption for DHM co-crystal formulations compared with the commercially available form of free DHM (Fig. 6c). Corresponding increases in DHM in brain tissues of dosed mice suggest that increased serum levels translate to higher levels of DHM crossing the blood–brain barrier in mice fed DHM co-crystal formulations (Fig. 6d). These data provide evidence for enhanced bioavailability by DHM co-crystal formulations compared with free DHM.

## Discussion

Despite tremendous overall H-bonding potential, DHM exhibits remarkable hydrophobic behavior at a macroscopic level. The hydrophobic tendency of DHM is explained by its dihydrate crystal structure, which shows phenols of DHM totally satisfying the H-bonding potentials of neighboring DHM molecules through an extensive network of H-bond pairings. In addition, the aromatic rings of DHM form favorable pi-stacking and Van der Waals interactions in the DHM hydrate, resulting in a stable form that is thermodynamically costly to solubilize in water. Our data establish that trolamine physically disrupts packing of DHM co-crystals and the H-bonding network. These data provide a physical and thermodynamic basis explaining the transformed macroscopic properties of DHM co-crystals, which exhibit up to five times increased water solubility. Co-formers or counterions replace and only partially satisfy the hydrogen bonding potential of DHM in the crystal, thereby leading to a more favorable thermodynamic state for solubilization. The cryptic hydrogen bonding potential that allows DHM to mask phenols by aggregation may also explain its tendency to better permeate the CNS compared with other polyphenols.

DHM has been investigated for its effects in mitigating alcohol use-related disorders, age-related diseases, oxidative stress, poisoning, and liver damage. However, its commercial form is practically insoluble in water, limiting its delivery and biopharmaceutical performance. Enhancing DHM’s physical properties to create a pharmaceutically acceptable formulation is thus of great interest. DHM, behaving as a Brønsted–Lowry acid–base with a pK_a_ in the useful pH range of 7–8, allows for feasible co-former or co-crystal formulations. Ionized species of DHM enable the preparation of various DHM co-crystal forms with bases that satisfy full or partial proton transfer, facilitating solid and liquid in situ extemporaneous pharmaceutical dosages. As illustrated in our model in Fig. S1^2^, DHM co-crystals described here have a composition that improves biopharmaceutical performance in a pharmaceutically acceptable context, which is expected to enable more accurate and efficient dosing by increasing solubility for better absorption of higher dose fractions.

As a possible tool in the management of AD, DHM holds potential in two possible realms. (ⅰ) DHM is a lead for medicinal chemistry structure-activity studies to generate pharmaceutical-grade anti-tau medications with improved potency and CNS permeability. (ⅱ) DHM co-crystals as dietary supplements offer a more immediate possible approach to combine with other lifestyle adaptations to aid healthier aging. Natural products are affordable and readily accessible chemicals, and in the case of DHM, could be rapidly developed as co-crystalline salt formations and deployed commercially and for research to support studies investigating healthier brain aging alongside complementary lifestyle modifications (i.e. attention to diet, exercise, and cardiovascular health). The MicroED structure shows that co-crystalline formulations of DHM remain unaltered in chemical structure despite improvements in aqueous solubility and apparent potency. Thus, the formulations we describe do not alter the natural product and GRAS status of DHM.

In vitro and in vivo PK data confirm higher kinetic solubilities, absorption, and distribution for co-crystalline formulations of DHM in SIF and mouse models. The trends mirrored the increased solubility patterns we observed for DHM co-crystals. Larger LogD values observed for DHM co-crystal formulations indicate enhanced partitioning into the hydrophobic phase, suggesting potential improvements in cell permeability. The phenomenon of increasing solubility and LogD trends could be driven by the augmented dissolution characteristics of DHM co-crystals. These co-crystals yield higher solute concentrations that, when solubilized, revert to the characteristics of the parent compound by re-aggregating, accounting for the suitability of dissolved DHM co-crystal formulations to re-partition into the hydrophobic phase.

The robust stability of DHM, DHM-TEA, and DHM-Ca(b) in both mouse and human LMs implies that DHM is not a significant substrate for most liver enzymes, including cytochrome P450s. Previous studies have indicated that DHM primarily undergoes phase II metabolism, involving processes like O-methylation, glucuronidation, and dihydroxylation, with limited information available about its interaction with phase I enzymes (14). It’s worth noting that DHM has been reported to inhibit certain CYP450s, and in this context, the DHM-Ca(b) formulation displays an intermediate half-life and rate of intrinsic clearance. It is possible that DHM’s remarkable stability in LMs may result from its inhibition of CYP450s (23), and CYP inhibition could be partially alleviated by formulating it as a calcium salt. The inability of DHM and DHM-TEA to be efficiently eliminated raises possible concerns about the potential for toxic accumulation, although the intermediate stability observed for calcium salt formulations might help mitigate potential adverse effects. The in vivo results from the DHM co-crystals are well aligned with the expected increase in bioavailability. Our studies conducted in mice did not demonstrate any adverse effects, and all formulations were well consumed and tolerated by mice at the doses tested. Our terminal DHM measurements represent the dynamic equilibrium achieved by reaching *C*_ss_ where the concentrations of DHM administered through crystallization technology remained consistently higher in the blood and brains of mice compared with free DHM.

Taken together, these data suggest that pharmacological properties can be finely tuned using various co-crystalline salt formulations to modulate PK stability. By strategically coupling structural and formulations chemistry, DHM was realized as a possible natural product inhibitor for targeting prionogenic seeding by AD tau. These studies open the door for near-term investigations of the effects of DHM salts as dietary supplements for testing in humans to support healthier aging, and in the broader context, suggest co-crystalline and/or salt forms may be extended to formulate other pseudo-hydrophobic medicinal chemicals to enhance solubility and efficiencies of dosing.

## Materials and methods

### In silico modeling

Molecular docking of DHM on PHF tau (PDB: 5O3L) was performed using Cavity-detection-guided Blind Docking (CB-Dock), a method developed by the Yang Cao Lab at Sichuan University that utilizes AutoDock Vina (19). The top five potential binding cavities were identified.

### Solubility determination

DHM co-crystal was dissolved to 10 mM in DMSO, and absorbance spectra were collected to identify the peak wavelength. The 10 mM stocks were diluted in a series (1:3 to 1:300), and absorbance was measured at the peak wavelength using the NanoDrop One/OneC Microvolume UV-Vis Spectrophotometer to produce a calibration curve. The working linear range was determined by excluding absorbance measurements above the limit of linearity. Stocks used for tau seeding assays were diluted to fall within the linear range, and their absorbances were used to calculate true stock concentrations.

### Differential scanning calorimetry

Samples (3–5 mg) were analyzed using a PerkinElmer DSC 8500 with a cooling system and continuous dry nitrogen purge at 25 mL/min. The instrument was calibrated with indium, tin, and lead samples. Data were collected from 25 to 300 °C at a heating rate of 10 °C/min. Onset and peak of thermal events were recorded and qualitatively analyzed for comparison.

### Cell culture and tau seeding

HEK293T cell lines stably expressing tau-K18CY (24) with GFP were cultured in DMEM with 10% FBS, 1% penicillin/streptomycin, and 1% glutamax at 37 °C in 5% CO_2_. For inhibitor testing, 0.2 g of cortical or hippocampal tissue was homogenized in sucrose buffer containing 1 mM EGTA and 5 mM EDTA using a Kinematica POLYTRON, as described previously (25). Homogenized brain was diluted 1:20 with Opti-MEM and sonicated for 3 min at 40% power. Brain homogenate incubated with inhibitor for 16 to 24 h at 4 °C to yield a final inhibitor concentration of 10 μM (on the biosensor cells) was sonicated prior to transfecting using Lipofectamine 2000 and Opti-MEM. After 20 min, 10 µL of inhibitor-treated fibrils were added to 100 µL of cells in triplicate. Seeded aggregates were quantified using the BioTek Cytation 5 Imaging Multimode Reader and ImageJ software. Seeded aggregates were counted and normalized to cell confluence as described in Figs. S3–S5 of Ref. (26). The average number of seeded aggregates per well, normalized to confluence, was plotted.

### MicroED

Refer to the Supplementary Text for details about sample preparation and data collection. The MicroED data were saved in MRC format and converted to SMV format using the mrc2smv software (https://cryoem.ucla.edu/microed). The converted frames were indexed and integrated by XDS (27). Then two datasets were scaled and merged using XSCALE (27, 28), and the intensities were converted to SHELX hkl format using XDSCONV (27, 28). The merged dataset can be ab initio solved by SHELXT (29) and refined by SHELXL (30) to yield the final MicroED structure.

### In vivo serum and brain exposure experiments

Sixty-four male and female wild-type C57BL/6 mice (Jackson Laboratories, Bar Harbor, ME, USA) were individually housed and acclimated in a 12-h light/dark cycle with temperature (22 °C) and humidity (40–60%) controlled conditions, where feed and water were available ad libitum. Following acclimation, mice were randomly assigned to eight different groups (*n* = 4/sex/group) where they were orally administered either a low dose (0.1 mg/mL) or a high dose (1.0 mg/mL); crystallized free DHM, DHM + TEA, or DHM + lysine co-crystals. For DHM + Ca(OH)_2_ Ca(OH)_2_, a solution of 0.5 mg/mL was used since higher concentration solution became cloudy over time. DHM was administered orally by dissolving the crystal powders into their drinking water where mice had continued ad libitum access to fluids. The study period lasted 10 days to observe the drinking patterns and general palatability of DHM crystals compared with free DHM and to ensure that steady-state concentrations of DHM were reached. Fluid and food intake along with body weights were measured daily. After the study period ended, mice were euthanized via CO_2_ exposure followed by cardiac puncture. Blood was collected and kept at room temperature in an EDTA-coated microcentrifuge tube, and serum was separated by centrifugation for 10 min at 5,000*×g* at 4 °C and stored at −80 °C until use. Whole brains were harvested and snap-frozen and stored at −80 °C until use. Protocols involving animals used in these studies were approved by the University of Southern California’s Department of Animal Resources Institutional Animal Care and Use Committee.

### LCMS

Brain and serum samples were prepared and extracted according to the procedure described by Carry et al. (14). Briefly, brain tissue was homogenized using a Misonix S-4000 Sonicator. Biological specimens were spiked with a 1 µg/mL isoquercetin standard solution in EtOAc to yield a final concentration of 0.5 µg/mL. Samples were dried using a Caliper TurboVap LV Evaporator and stored at −80 °C prior to reconstitution and analysis. LC-MS data was conducted using binary mobile phases, with conditions replicated from Carry et al. (14): phase A was water with 0.1% formic acid (FA), phase B was acetonitrile with 0.1% FA, the flow rate was 0.450 mL/min, the column temperature was 30 °C, and the autosampler temperature was 4 °C. Given that DHM was the analyte of interest, the LC gradient used for chromatographic separation was initially replicated and then optimized using DHM powder dissolved in methanol (1 µg/mL). The gradient run included diversions: the first 4.5 min of elution were led to waste (co-crystals, nontarget eluents), the next 3 min of elution were led to the mass spectrometer (retention time from 4.5 to 7.5 min where DHM and the IS elute), followed by 3 min of equilibration. Samples and standards were injected at a volume of 3 µL. Analyte abundances (DHM and IS) were determined in negative ionization mode and quantified from peak areas by multiple reaction monitoring.

## Supplementary Material

pgaf007_Supplementary_Data

## Data Availability

MicroED structure data for DHM co-crystals with trolamine may be downloaded from the Cambridge Structural Database, CCDS accession code 2415045.

## Preprints

This manuscript was posted on a preprint at: https://www.biorxiv.org/content/10.1101/2023.04.25.538327v1.

## References

[1] Savjani KT, Gajjar AK, Savjani JK. 2012. Drug solubility: importance and enhancement techniques. ISRN Pharm. 2012:195727. 10.5402/2012/195727 22830056 PMC3399483

[2] Amidon GL, Lennernäs H, Shah VP, Crison JR. 1995. A theoretical basis for a biopharmaceutic drug classification: the correlation of in vitro drug product dissolution and in vivo bioavailability. Pharm Res. 12:413–420. 7617530 10.1023/a:1016212804288

[3] Elder DP, Holm R, Diego HLD. 2013. Use of pharmaceutical salts and cocrystals to address the issue of poor solubility. Int J Pharm. 453:88–100. 23182973 10.1016/j.ijpharm.2012.11.028

[4] Shi D, Nannenga BL, Iadanza MG, Gonen T. 2013. Three-dimensional electron crystallography of protein microcrystals. eLife. 2:e01345. 24252878 10.7554/eLife.01345 PMC3831942

[5] Nannenga BL, Shi D, Leslie AGW, Gonen T. 2014. High-resolution structure determination by continuous-rotation data collection in MicroED. Nat Methods. 11:927–930. 25086503 10.1038/nmeth.3043 PMC4149488

[6] Seidler PM, et al 2022. Structure-based discovery of small molecules that disaggregate Alzheimer's disease tissue derived tau fibrils in vitro. Nat Commun. 13:5451. 36114178 10.1038/s41467-022-32951-4 PMC9481533

[7] Chen L, et al 2021. Dihydromyricetin acts as a potential redox balance mediator in cancer chemoprevention. Mediators Inflamm. 2021:6692579. 33776577 10.1155/2021/6692579 PMC7979283

[8] Silva J, et al 2020. Dihydromyricetin improves mitochondrial outcomes in the liver of alcohol-fed mice via the AMPK/Sirt-1/PGC-1α signaling axis. Alcohol. 91:1–9. 33080338 10.1016/j.alcohol.2020.10.002 PMC7902334

[9] Chen S, et al 2015. Dihydromyricetin improves glucose and lipid metabolism and exerts anti-inflammatory effects in nonalcoholic fatty liver disease: a randomized controlled trial. Pharmacol Res. 99:74–81. 26032587 10.1016/j.phrs.2015.05.009

[10] Chu J, et al 2018. Dihydromyricetin relieves rheumatoid arthritis symptoms and suppresses expression of pro-inflammatory cytokines via the activation of Nrf2 pathway in rheumatoid arthritis model. Int Immunopharmacol. 59:174–180. 29656207 10.1016/j.intimp.2018.04.001

[11] Jing N, Li X. 2019. Dihydromyricetin attenuates inflammation through TLR4/NF-kappaB pathway. Open Med (Wars). 14:719–725. 31572805 10.1515/med-2019-0083 PMC6749725

[12] Dong S, Ji J, Hu L, Wang H. 2019. Dihydromyricetin alleviates acetaminophen-induced liver injury via the regulation of transformation, lipid homeostasis, cell death and regeneration. Life Sci. 227:20–29. 30974116 10.1016/j.lfs.2019.04.019

[13] Silva J, et al 2020. Dihydromyricetin protects the liver via changes in lipid metabolism and enhanced ethanol metabolism. Alcohol Clin Exp Res. 44:1046–1060. 32267550 10.1111/acer.14326 PMC7211127

[14] Carry E, et al 2021. Identification of dihydromyricetin and metabolites in serum and brain associated with acute anti-ethanol intoxicating effects in mice. Int J Mol Sci. 22:7460. 34299083 10.3390/ijms22147460 PMC8307506

[15] Silva J, Yu X, Qi L, Davies DL, Liang J. 2020. Antialcohol effects of dihydromyricetin in combination with other flavonoids. Nat Prod Commun. 15:2–6.

[16] Guan D, et al 2021. Improving the physicochemical and biopharmaceutical properties of active pharmaceutical ingredients derived from traditional Chinese medicine through cocrystal engineering. Pharmaceutics. 13:2160. 34959440 10.3390/pharmaceutics13122160 PMC8704577

[17] U.S. Food and Drug Administration . Inactive Ingredients Database Download. 2022 [accessed 2023 Mar 23]. https://www.fda.gov/drugs/drug-approvals-and-databases/inactive-ingredients-database-download.

[18] Evaluation, A. S. F. P. A . National Plan to Address Alzheimer's Disease. [accessed 2023 Mar 23]. https://aspe.hhs.gov/reports/national-plan-2021-update#goal-6.

[19] Cao Y, Li L. 2014. Improved protein–ligand binding affinity prediction by using a curvature-dependent surface-area model. Bioinformatics. 30:1674–1680. 24563257 10.1093/bioinformatics/btu104

[20] Xu Z, Liu B, Ning Z, Zhang Y. 2007. Racemic dihydromyricetin dihydrate. Acta Crystallographica Section E. 63:o4384–o4384.

[21] Matthews BW . 1968. Solvent content of protein crystals. J Mol Biol. 33:491–497. 5700707 10.1016/0022-2836(68)90205-2

[22] Ghose AK, Herbertz T, Hudkins RL, Dorsey BD, Mallamo JP. 2012. Knowledge-based, central nervous system (CNS) lead selection and lead optimization for CNS drug discovery. ACS Chem Neurosci. 3:50–68. 22267984 10.1021/cn200100h PMC3260741

[23] Liu L, Sun S, Rui H, Li X. 2017. In vitro inhibitory effects of dihydromyricetin on human liver cytochrome P450 enzymes. Pharm Biol. 55:1868–1874. 28614988 10.1080/13880209.2017.1339284 PMC7012011

[24] Holmes BB, et al 2014. Proteopathic tau seeding predicts tauopathy in vivo. Proc Natl Acad Sci U S A. 111:E4376–E4385. 25261551 10.1073/pnas.1411649111 PMC4205609

[25] Seidler PM, et al 2019. Structure-based inhibitors halt prion-like seeding by Alzheimer’s disease-and tauopathy-derived brain tissue samples. J Biol Chem. 294:16451–16464. 31537646 10.1074/jbc.RA119.009688 PMC6827308

[26] Seidler PM, et al 2018. Structure-based inhibitors of tau aggregation. Nat Chem. 10:170–176. 29359764 10.1038/nchem.2889 PMC5784779

[27] Kabsch W . 2010. XDS. Acta Crystallogr D Biol Crystallogr. 66:125–132. 20124692 10.1107/S0907444909047337 PMC2815665

[28] Kabsch W . 2010. Integration, scaling, space-group assignment and post-refinement. Acta Crystallogr D Biol Crystallogr. 66:133–144. 20124693 10.1107/S0907444909047374 PMC2815666

[29] Sheldrick GM . 2015. SHELXT—integrated space-group and crystal-structure determination. Acta Crystallogr A Found Adv. 71:3–8. 25537383 10.1107/S2053273314026370 PMC4283466

[30] Sheldrick GM . 2015. Crystal structure refinement with SHELXL. Acta Crystallogr C Struct Chem. 71:3–8. 25567568 10.1107/S2053229614024218 PMC4294323

