# Supplementary material for: Improving the solubility of pseudo-hydrophobic chemicals through co-crystal formulation
Source: PNAS Nexus. 2025 Jan 13;4(1):pgaf007. doi: 10.1093/pnasnexus/pgaf007 (PMC11756387; doi:10.1093/pnasnexus/pgaf007)
Supplement: pgaf007_Supplementary_Data [file pgaf007_supplementary_data.pdf]

Improving the solubility of pseudo-hydrophobic chemicals through co-crystal formulation

Isis Janilkarn-Urena[1,9], Amanda Tse[1,9], Jieye Lin[3,4,7,9], Bliss Tafolla-Aguirre[1,9] , Alina Idrissova[1], Mindy Zhang[1], Samantha G. Skinner[2], Nader Mostowfi[1], Jinah Kim[1], Nikhila Kalapatapu[2], Xinmin Chang[1], Christina Efthymiou[1], Christopher K. Williams[6], Shino D. Magaki[6], Harry V. Vinters[6,7], Tamir Gonen[3,4,5], S. Kaleem Ahmed[8], Hovhannes J. Gukasyan[1,10], Daryl L. Davies[2], Paul M. Seidler[1,10]

1 Department of Pharmacology and Pharmaceutical Sciences, University of Southern California Mann School of Pharmacy and Pharmaceutical Sciences, 1985 Zonal Ave, Los Angeles, CA 90089-9121, USA. 2 Titus Family Department of Clinical Pharmacy, University of Southern California Mann School of Pharmacy, Los Angeles, CA 90089, USA. 3 Department of Biological Chemistry, University of California Los Angeles, 615 Charles E. Young Drive South, Los Angeles, CA 90095, USA. 4 Howard Hughes Medical Institute, University of California Los Angeles, Los Angeles, CA 90095, USA. 5 Department of Physiology, University of California Los Angeles, 615 Charles E. Young Drive South, Los Angeles, CA 90095, USA. 6 Department of Pathology and Laboratory Medicine, David Geffen School of Medicine at University of California, Los Angeles, California 90095. 7 Department of Neurology, David Geffen School of Medicine at University of California, Los Angeles, California 90095. 8 University of Southern California Medicinal Chemistry Core Laboratory at the Alfred E. Mann School of Pharmacy and Pharmaceutical Sciences, Los Angeles, California 90095. 9 Authors contributed equally to experimental work.10 Correspondence: pseidler@usc.edu; Tel.: +1-323-442-2574.

SUPPLEMENTAL TEXT

Materials

A commercially available batch of dihydromyricetin [(DHM) IUPAC Name (2R,3R)-3,5,7-trihydroxy-2-(3,4,5-trihydroxyphenyl)-2,3-dihydrochromen-4-one, of ≥ 98% purity] was purchased from Master Herbs Inc (Pomona, CA). Counterions, triethanolamine (99%) was purchased from Lab Alley (Spicewood, TX); sodium hydroxide, calcium hydroxide, and L-Lysine were all purchased from Sigma-Aldrich (Burlington, MA); and TRIS-base was purchased from Gold Biotechnology (St. Louis, MO). Solvents, DMSO, pure ethyl alcohol (200 proof, molecular grade), methanol (≥99.5% purity, molecular grade), 2-propanol (≥99.5% purity, molecular grade), acetone (≥99.5% purity, molecular grade), and anti-solvent, chloroform (anhydrous, ≥99%, containing 0.5-1.0% ethanol as stabilizer) were all purchased from Sigma-Aldrich (Burlington, MA).

For DHM Quantification, the following materials and instrumentation were procured for use. L-ascorbic acid (99% pure), Ethyl acetate (>99.5% pure), and formic acid (>95% pure) were purchased from Sigma-Aldrich (St. Louis, MO). HPLC-grade methanol, HPLC-grade water, and HCl (6.0 N) were purchased from VWR (Radnor, PA). The internal standard, isoquercetin, was purchased from MedChemExpress (Monmouth Junction, NJ) at 99.87% purity. An Agilent 1260 Infinity LC System (Agilent Technology, Palo Alto, CA, USA) and Sciex Triple Quad 6500+ System (Carlsbad, CA, USA) comprised the LC-MS set up for biological sample analysis. The Waters Acquity UPLC BEH C8 Column (2.1x150 mm, 1.7 um) (Milford, MA, USA) equipped with a VanGuard Acquity C8 guard column (2.1x5mm, 1.7 um) (Milford, MA, USA) was utilized for chromatographic separation of analytes present in the processed mouse serum and brain samples.

pKa Determination

pKa values were calculated using ADMET Predictor™ version 10.3.0.7 64-bit edition module of Simulations Plus software (Lancaster, CA), and ChemDraw® Professional version 20.1.1.125 (PerkinElmer Informatics, Inc.). Potentiometric titration curves of DHM were obtained by adding 0.1mL of a 0.5N NaOH standard solution to three different 2mg/mL DHM analyte solutions. All solutions were made in 50mL beakers using deionized water and prepared at 22-25˚C. The 2mg/mL DHM standard solution was titrated with 0.5N NaOH. The NaOH standard solution was prepared by dissolving 3g of sodium hydroxide in 150mL of water for a final concentration of 0.5N NaOH. A 100mg/mL stock solution of DHM was prepared by weighing 0.5g of DHM using an analytical balance and dissolving it in 5mL of methanol in a 15mL conical tube. Three analyte solutions were prepared using methanol and water mixtures to yield 25mL of solutions with 25, 50, and 75% v/v methanol content. Five hundred µL of DHM stock solution was then added to the three analyte solutions to yield final DHM concentration of 2mg/mL, the initial pH value was measured and recorded.

pKa was orthogonally determined through UV-VIS spectroscopy optical absorption technique described by Pandey, et. al.[32] that was adapted and modified. Briefly, a 5mM analyte stock solution of DHM was prepared by measuring 16 mg of DHM dissolved in 10mL of DMSO and was further diluted to 2mM and used as the working stock solution. Buffer solutions of various pH values were prepared as described in Supplementary Table 1. Using a 96-well plate, 0.2mL of each pH buffer solution was added to duplicate wells, then 0.5µL/well of the DHM stock solution was added for a final DHM concentration of 5µM in each sample well. A spectral scan was conducted (λ = 230-700nm), and absorbance was measured in 10nm increments. The pKa was calculated using $pKa=pH+\log [(DHM^{0}_{abs}-DHM^{pH}_{abs})/(DHM^{pH}_{abs}-DHM^{-}_{abs})$, where DHM^{0}_{abs} is the absorbance of the unionized molecule at lowest pH, DHM^{pH}_{abs} is the absorbance of the molecule in respective buffers tested, and DHM^{-}_{abs} is the absorbance of the ionized molecule at highest pH.

Counterion Screen

Various counterions indicated in the main text were screened by preparation in a variety of solvents (methanol, ethanol, 2-propanol, or acetone) to facilitate crystallization with DHM by slow evaporation. Crystallization was carried out at ambient temperature (22-25˚C) in 250mL Erlenmeyer flasks. A 1:1, 1:1.25, or 1:2 molar ratio of DHM to counterion were tested to find optimum stoichiometry for crystallization. The counterion was first dissolved in the respective solvent and DHM added and to it with stirring for at least 45 min until homogenous slurries were observed. Slurries were then shielded from light and allowed to slowly evaporate for two weeks. Resulting powders were washed with 50mL of chloroform over a vacuum pump membrane filtration setup. Filtrate residue was dried, recovered from filter membrane, and stored in sealed glass vials at ambient conditions further characterization and biological activity testing.

X-Ray Powder Diffractometry

Solid material isolated from DHM slurries was ground into a fine powder using a glass coverslip or a spatula. Approximately 5-10 mg of the powder was placed into a zero background silicone sample holder (5mm diameter × 0.2mm deep, Rigaku, Inc., The Woodlands, TX). Analysis was performed using a Rigaku Miniflex 600 Benchtop XRD System with a scanning rate of 2° min^{-1}. Diffractograms were recorded at diffraction angles (2θ) from 0° to 50° at room temperature.

Quantitative EM (qEM) imaging

AD tau fibrils were purified by homogenizing AD brain tissues in sucrose buffer with 1 mM EGTA and 5 mM EDTA. The crude brain extracts were heated to 95°C for 20 minutes to precipitate proteins. The homogenates were then centrifuged at 20,100 x g for 30 minutes at 4°C, and the supernatants were ultracentrifuged at 95K for one hour. The ultrapellets containing purified fibrils

were resuspended in 1X PBS, pH 7.4. Purified AD brain-derived tau fibrils were diluted 1:10 in PBS and incubated with EGCG or DHM ligands for 48 hours at 4°C. Negatively stained EM grids were prepared by depositing 6 µl of fibril samples on formvar/carbon-coated copper grids (400 mesh) for 3 minutes, followed by rapid blotting and staining with 4% uranyl acetate for 2 minutes. Grids were wicked dry with filter paper. For qEM, negatively stained grids were screened using a JEOL 2100 TEM at ×12,000 magnification, collecting 99 images in consistent increments. Visible fibrils were manually counted and analyzed in triplicate groups of 33 micrographs for each experimental condition.

In vivo serum and brain exposure experiments

Sixty-four male and female wild-type C57BL/6 mice (Jackson Laboratories, Bar Harbor, ME, USA) were individually housed and acclimated in 12 h light/dark cycle with temperature (22° C) and humidity (40-60%) controlled conditions, where feed and water were available ad libitum. Following acclimation, mice were randomly assigned to eight different groups (n=4/sex/group) where they were orally administered either a low dose (0.1 mg/mL) or a high dose (1.0 mg/mL); crystallized free DHM, DHM + TEA, or DHM + lysine co-crystals. For DHM + $Ca(OH)_2$ $Ca(OH)_2$ a solution of 0.5 mg/mL was used since higher concentration solution became cloudy over time. DHM was administered orally by dissolving the crystal powders into their drinking water where mice had continued ad libitum access to fluids. The study period lasted 10 days to observe the drinking patterns and general palatability of DHM crystals compared to free DHM, and to assure that steady state concentrations of DHM were reached. Fluid and food intake, along with body weights were measured daily. After the study period ended, mice were euthanized via CO2 exposure followed by cardiac puncture. Blood was collected and kept at room temperature in a EDTA coated microcentrifuge tube and serum was separated by centrifugation for 10 min at 5,000 x g at 4° C and stored at -80 °C until use. Whole brains were harvested and snap-frozen and stored at -80° C until use. Animals used in the study were considered and handled in adherence to the University of Southern California's Department of Animal Resources Institutional Animal Care and Use Committee (IACUC) policies and guidelines.

MicroED sample preparation, data collection and processing.

The DHM-TEA co-crystal was prepared for MicroED as described previously.[33] Around 1 mg ground powder was transferred into a 10mL scintillation vial and mixed with a carbon-coated copper grids (400-mesh, 3.05 mm O.D., Ted Pella Inc.) which was pretreated with glow-discharge plasma at 15 mA for 60 s on the negative mode using PELCO easiGlow (Ted Pella Inc.). After a gentle shaking of the vial, the grid was taken out and clipped at room temperature.

The clipped grid was loaded in an aligned Thermo Fisher Talos Arctica Cryo-TEM (200 kV, ~0.0251 Å) at 100 K, equipped with a CetaD CMOS camera (4096 × 4096 pixels). Screening of size-suitable microcrystals was done in the imaging mode (SA 3400×). The MicroED data was collected in the diffraction mode with 829 mm diffraction length, 70µm C2 aperture and a 100µm selected area aperture in the parallel beam condition (45.2% C2 intensity) which resulted a beam size at approximately 2.5 µm. Typical data collection was performed using a constant rotation rate of ~1 deg/s over an angular wedge of 130° from -65° to +65°, with 1s exposure time per frame. Crystals as selected for MicroED data collection were isolated and calibrated to eucentric height to maintain the crystal inside the beam during the rotation.

Kinetic solubility determination in simulated intestinal fluid (SIF)

SIF was prepared by dissolving 6.8 g of KH_2PO_4 into 500 mL ultrapure water and pH adjusted to 6.8 with NaOH (Buffer A). A second buffer, Buffer B, was prepared by dissolving 10 g of pancreatin

powder in 400 mL of ultrapure water. Buffers A and B were mixed and diluted with ultrapure water to 1000 mL at room temperature to achieve a final pancreatin concentration of 10 mg/mL at pH 6.79. Stock solutions of DHM, DHM-TEA, and DHM-Ca(b) were prepared in DMSO at 10 mM concentrations. Diclofenac sodium (positive control) was prepared in DMSO at a 30 mM concentration.

30 µL stock solution of each compound was dispensed in a 96-well block followed by addition of 970 µL of SIF. Samples were prepared in duplicate. The solubility sample plate was mixed with stir bars and transferred to a thermomixer for 2 hour incubation at room temperature with shaking at 1,100 rpm. Supernatant were obtained after shaking incubation by centrifugation at 3220 g at ambient for 30 minutes, and a 50 µL aliquot of supernatant was removed. Solublility of remaining sample in supernatant was determined by LC-MS/MS relative to concentration standards for each given chemical of interest.

LogD determination

Stock solutions of DHM, DHM-TEA, and DHM-Ca(b) were prepared in DMSO at 10 mM concentrations. 15 µL of each was placed into a 96-well rack, and 500 µL of 1-octanol saturated phosphate buffer (pH 7.4) was added into each vial of the LogD plate followed by the addition of 500 µL of phosphate buffer saturated with 1-octanol. Samples were prepared in duplicate. LogD plates was shaken in a Eppendorf Thermomixer Comfort at ambient at 1,100 rpm for 1 hour. The samples were then centrifuged at RT at 20,000 g for 20 minutes, and the upper (1-octanol) and lower (buffer) phases were removed. For the upper phases, aliquots of 5 µL were removed and 495 µL of methanol/ultrapure water (50%/50%) added. 20 µL of the mixture was diluted with 180 µL of methanol/ultrapure water (50%/50%). For the lower phases, aliquots of 50 µL were removed and mixed with 450 µL of methanol/ultrapure water (50%/50%). Sample concentration was determined by LC-MS/MS.

Metabolic stability in pooled human and male mouse liver microsomes

A master solution was prepared with final concentrations of 100 mM phosphate buffer, 5 mM $MgCl_2$, and 0.5 mg/mL microsomes. NADPH solution was added to each sample for a final concentration of 1 mM in duplicate. NADPH was used as an enzyme co-factor to initiate Phase I metabolism reaction. Verapamil (positive control), DHM, DHM-TEA, or DHM-Ca(b) were added at a 1 µM final concentration. 50 µL aliquotes were removed from the reaction solution at 0, 15, 30, 45 and 60 minutes. The reaction was stopped by adding 4 volumes of cold acetonitrile with IS (100 nM alprazolam, 200 nM imipramine, 200 nM labetalol and 2 µM ketoprofen). Samples were centrifuged at 220 x g for 40 minutes and 90 µL aliquots of the supernatant was diluted with ultra-pure water and used for concentration determination by LC-MS/MS. Note: If remaining percentage at 60 minutes was greater than 85%, then $T_{1/2}$ and CL_{int} is reported as ">255.85" and "<5.42", respectively. If remaining percentage at 15 minutes was lower than 10%, then CL_{int} and $T_{1/2}$ is reported as ">307.01" and "<4.51", respectively.

LCMS parameters for identification of DHM

The mass transitions of interest were monitored for three minutes from an RT between 4.5 and 7.5 minutes with a dwell time of 200 ms. The following mass spectrometry parameters were optimized from the starting values mentioned in Fan et al., 2017[34]: GS1=55 psi, GS2=25 psi, CUR=45 psi, Collision gas=7, TEM=550 ^{o}C, IonSpray=4500, DP=-114.0 V, CE=-16.7 V for the mass transition (319→193), CE=-29.4 V for the mass transition (319→125), EP=10, CXP=-10 V.[2] The following mass spectrometer parameters for the internal standard (mass transition of

462.9→300) were gathered from He et al., 2013[35]: DP=-70 V, CE=-40 eV, CXP=-10 V, EP=10. The parameter values for the ESI capillary voltage, nozzle voltage, nebulizer gas (N_2) pressure, dry gas temperature/flow rate, and sheath gas temperature/flow rate remained consistent with those used in Carry et al., 2021[14]. Acronym Key**:** GS1 is source gas 1, GS2 is source gas 2, CUR is curtain gas, TEM is the temperature of the heaters applied to GS2, DP is declustering potential, CE is collision energy, EP is entrance potential, CXP is the collision cell exit potential.

Multiple reaction monitoring (MRM) mode was utilized to identify two main mass transitions for DHM and one mass transition for IS. The mass transitions for DHM were labeled as DHM quantifier (319→193) and DHM qualifier (319→125). The mass transition for the IS was 462.9→300. The presence of DHM was identified via the DHM quantifier mass transition (319→193) and the IS was identified via its single mass transition, in addition to their characteristic retention times of 5.83 and 6.74 respectively. Integration of the corresponding analyte peaks produced peak area values that were then compared between treatment groups to reveal general trends among the data. The chromatograms generated from running the biological samples are compiled in the supplementary material section. Isoquercetin was used as the internal standard (IS) at a final concentration of 500 ng/mL in all samples and standards.

SUPPLEMENTARY FIGURES

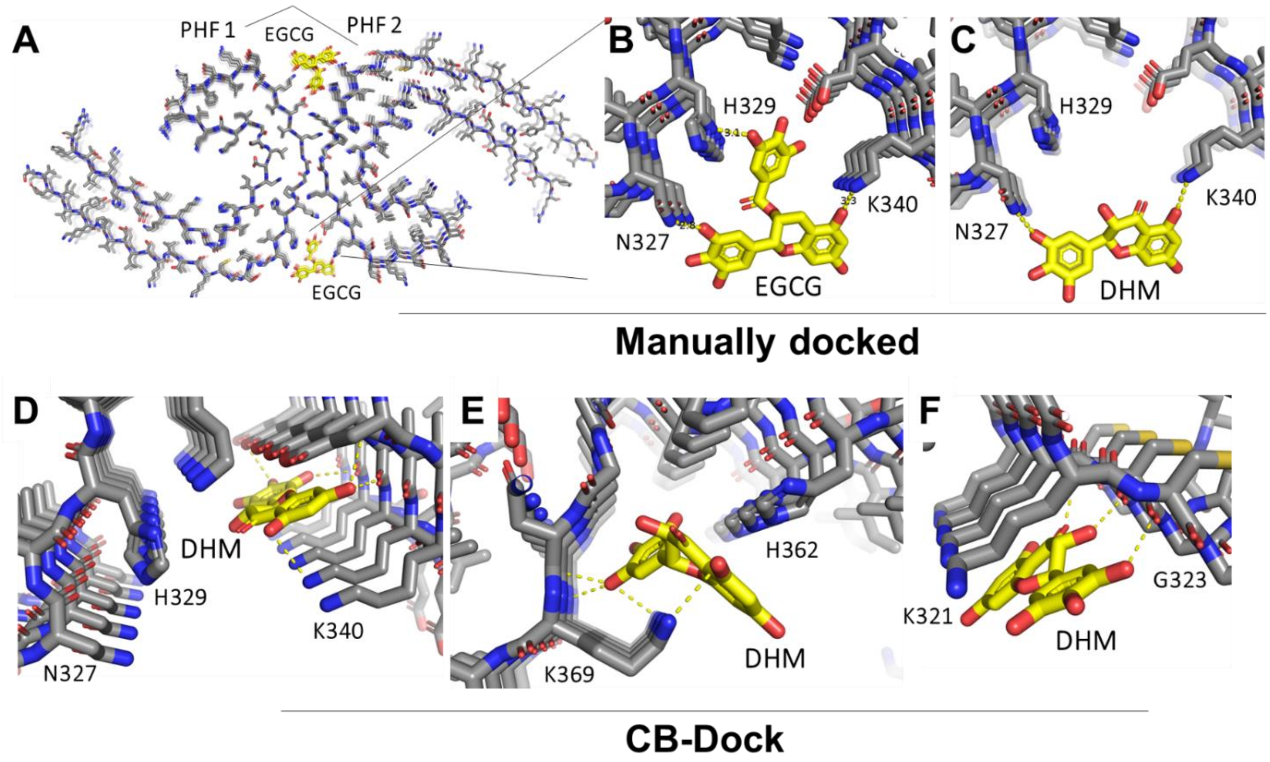

Supplementary Figure 1. (A and B) CryoEM structure of AD brain-derived tau PHF bound by EGCG. (C) Manually generated model of DHM binding to EGCG binding site of AD tau made by superimposing DHM with bound EGCG. (D-F) Alternative predicted DHM binding sites generated by CB-Dock. (D) Site 1 overlaps with the binding volume predicted in the manually generated model in C, except bound DHM is predicted to orient with aromatic rings perpendicular to the fibril axis burying in a cavity between Lys340 and Glu338. (E) Site 2 of DHM binding predicted by CB-Dock occurs in a cavity lined by His362 and K369. (F) Site 3 predicted by CB-Dock occurs with DHM packing aromatic moieties with the aliphatic chain of K321, and H-bonding occurring between the phenolic moieties and the amide backbone at a sharp turn formed by G323.

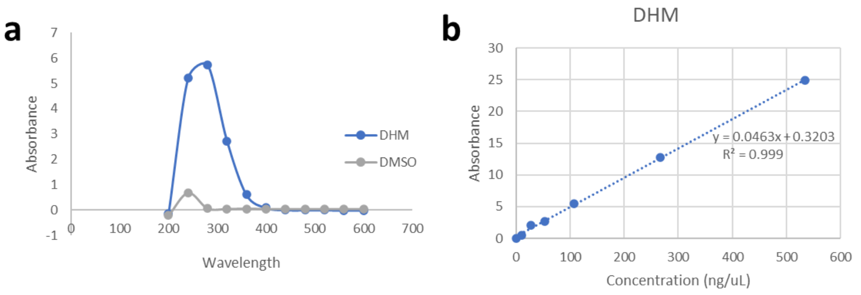

c

	DHM Solubility Limit (g/L)
**Dissolution Attempt 1 (37°C; 8,000 rpm)**	0.237
**Dissolution Attempt 2 (37°C; 15,000 rpm)**	0.609
**Dissolution Attempt 3 (room temp.; 8,000 rpm)**	0.652

Supplementary Figure 2. Aqueous solubility determination of DHM. (a) Absorbance scan of DHM dissolved in DMSO shows a peak at 280 nm. (b) Standard curves of DHM supernatants dissolved in water to a target concentration 10 mM and clarified by centrifugation. (c) Table detailing concentrations measured from DHM supernatants dissolved in water and determined from standard curve in b.

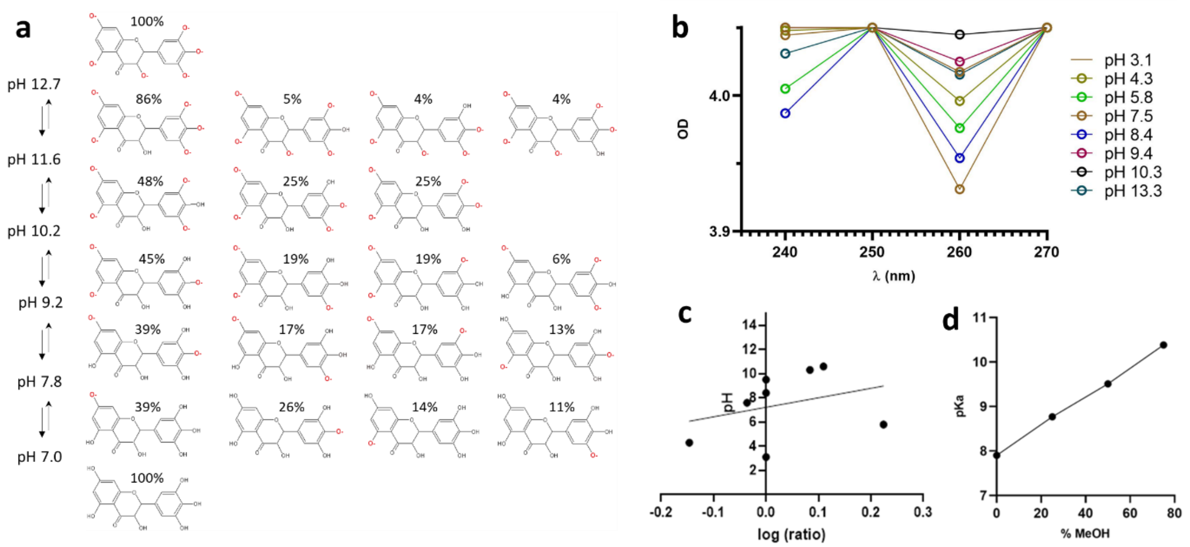

Supplementary Figure 3. Predicted and experimental pKa's of DHM. (a) Ionizations occurring from neutral to anionic states produced using ADMET Predictor™. Predicted proportions of respective ionized states are indicated. Microstates for multiply ionized phenols are shown as a function of increasing pH. (b and c) UV-Vis spectroscopy[31] pKa determination of DHM determined at pH values ranging from pH 3.1-13.3. (d) NaOH potentiometric titration curves with MeOH revealing an equivalence point at ~pH 7.9.

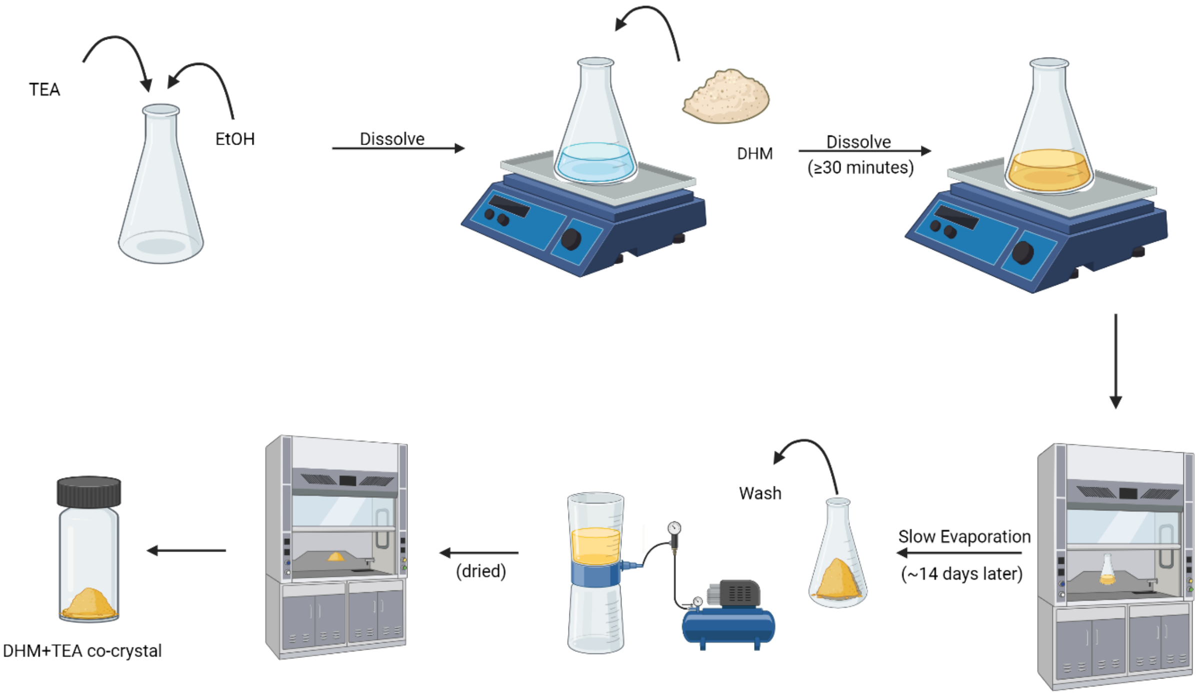

Supplementary Figure 4. Example of a slurry preparation using DHM and TEA. As shown, mixture was achieved by preparing a solution containing 0.41g of TEA dissolved in 75mL of ethyl alcohol (200 proof) on a magnetic stir plate. Once dissolved, 1.0g of DHM powder (1:1 molar ratio) was added to the solution containing TEA and stirred for at least 45 minutes. Precipitate recovered 14 days later was placed in a membrane filtration apparatus and vacuum washed with chloroform to remove possible remaining excess chloroform-extractable material, and then dried. After drying, solids were stored in a clear glass scintillation vial at room temperature (22-27˚C) until future use.

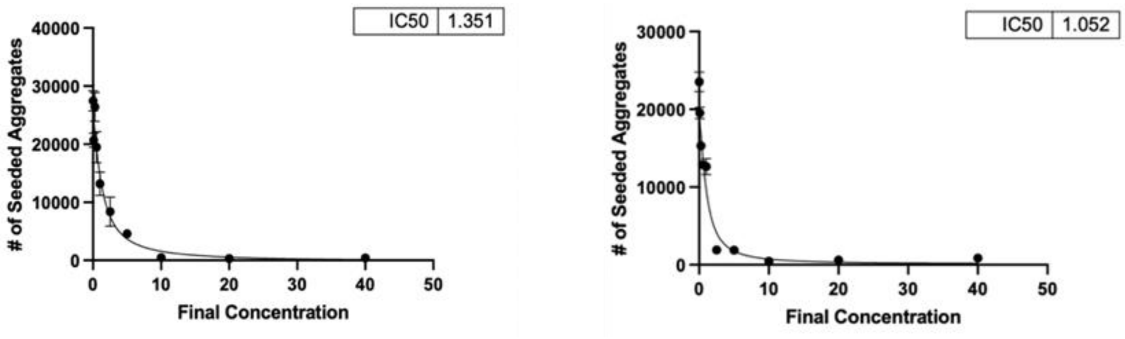

Supplementary Figure 5. IC50 curves generated as described in the main text, except using DHM-TEA and -Ca(b) formulations (left and right, respectively) dissolved in DMSO. Both formulations have IC50s similar to DHM when dissolved in DMSO (Fig. 1B main text), thus suggesting DHM is the active component in tau inhibitor assays and that counterions themselves do not exhibit inhibitory activity towards tau seeding.

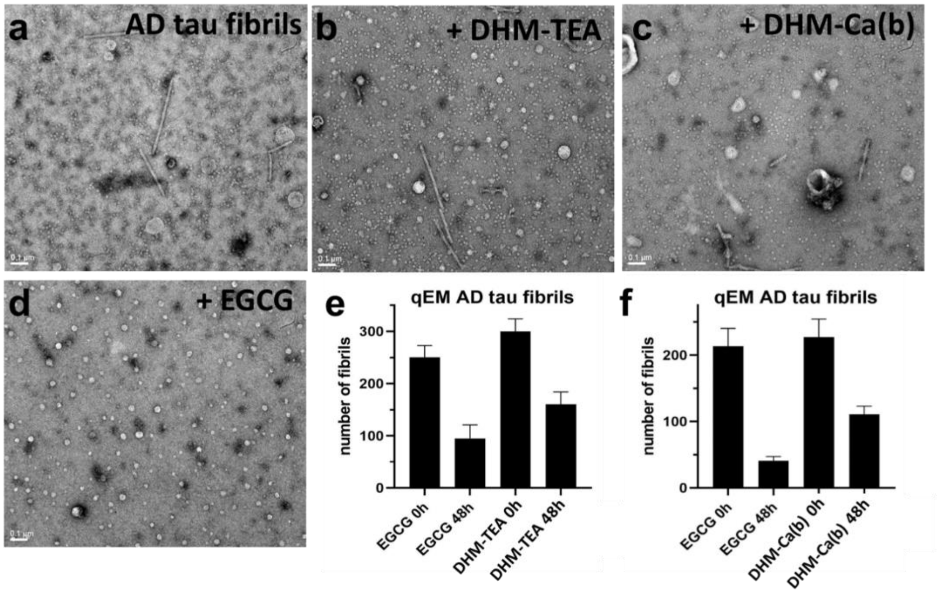

Supplementary Figure 6. (A) DHM-mediated AD tau fibril disaggregation, measured by qEM. Fibrils were counted from N=90 randomly acquired EM images, which were split three ways and quantified in triplicate. Columns show numbers of fibrils counted as a function of inhibitor pre-incubation time, as indicated. Error bars represent standard deviations. (B-D) Example EM images used from qEM after 48 hr inhibitor incubation.

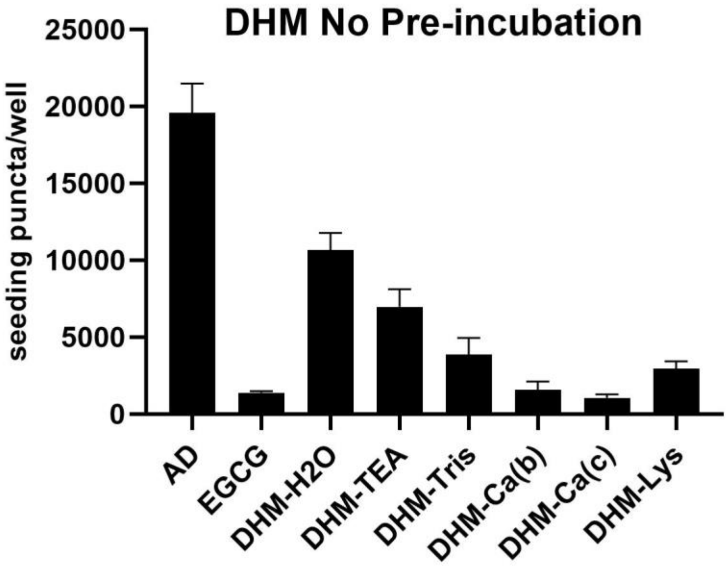

Supplementary Figure 7. Seeding inhibition measured as described in the main text, without inhibitor pre-incubation with crude AD brain homogenates.

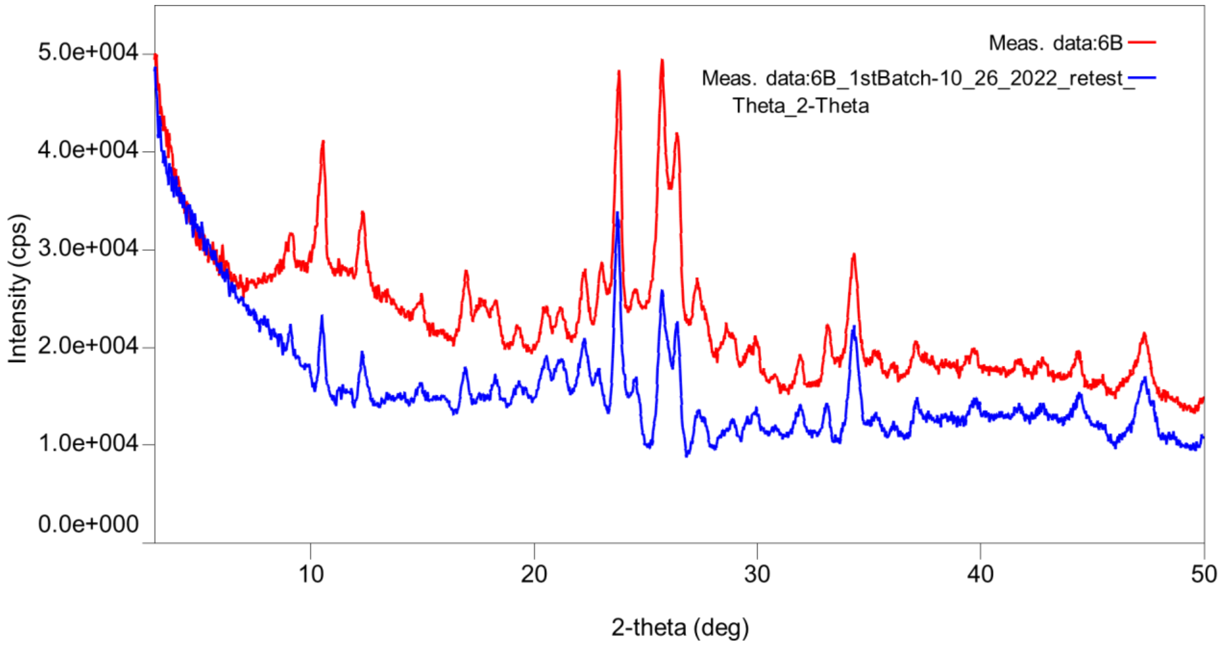

Supplementary Figure 8. X-Ray powder diffractogram comparing DHM-Ca salt b freshly prepared (red) overlayed with the same preparation aged 1 year at room temperature (blue).

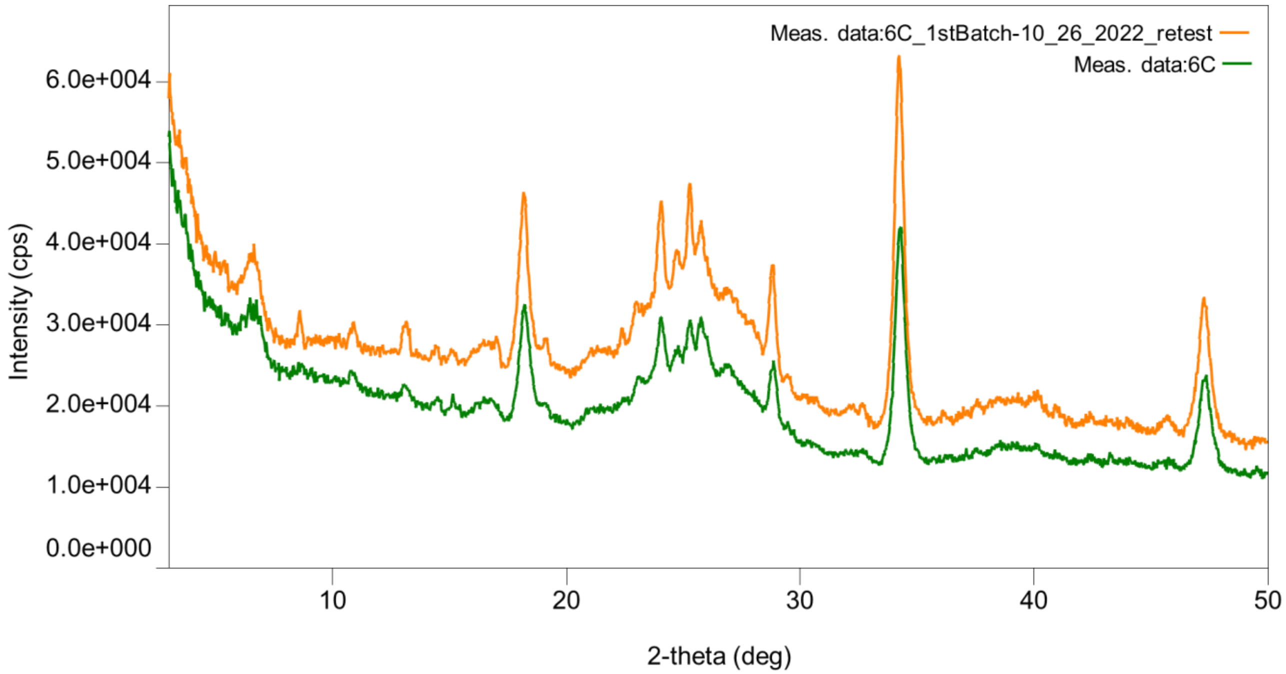

Supplementary Figure 9. X-Ray powder diffractogram comparing DHM-Ca salt c freshly prepared (orange) overlayed with the same preparation aged 1 year at room temperature (green).

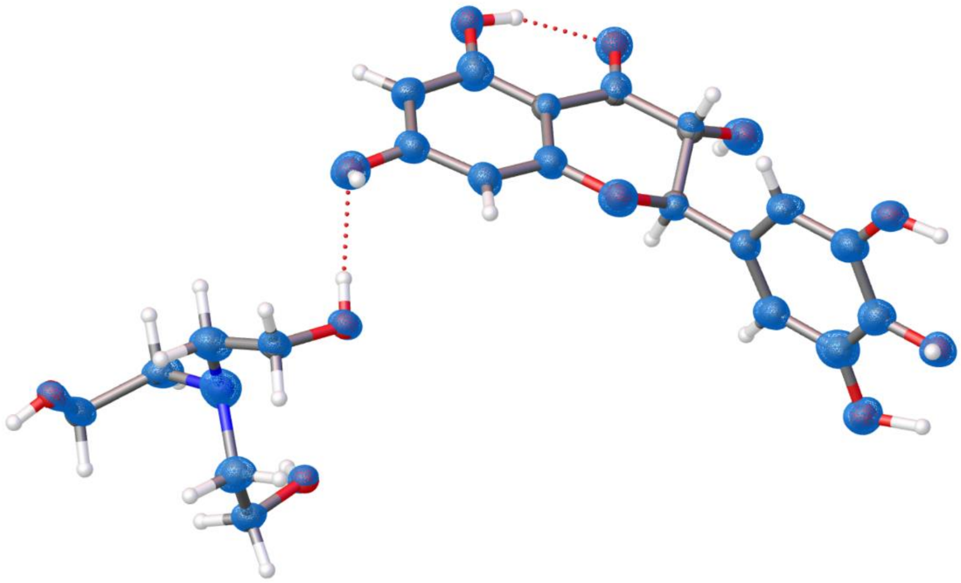

Supplementary Figure 10. The crystal structure of DHM-TEA as solved with a resolution of 0.75 Å. The blue mesh are $2F_o$-F_c electrostatic potential maps at the level of 0.67 e·Å^{-3}.

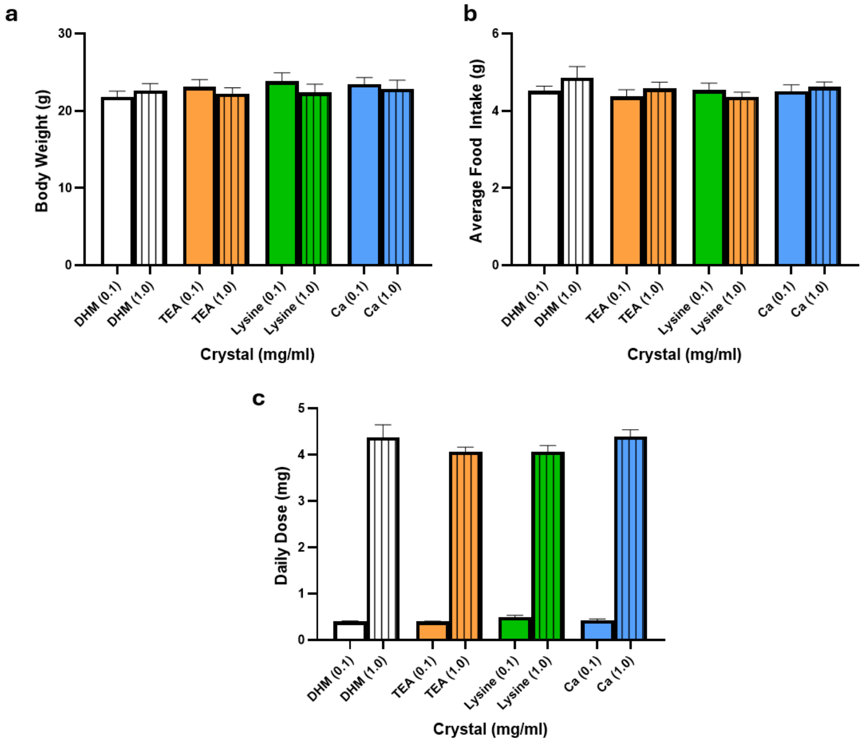

Supplementary Figure 11. Graphs showing **a)** average body weights of mice throughout the 10-day study period and **b)** average food intake of mice throughout the 10-day study period.

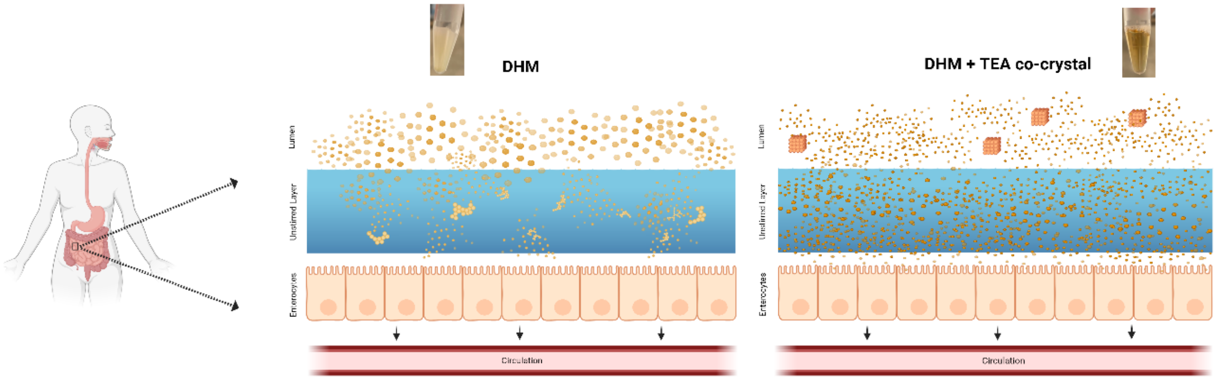

Figure 12. Proposed model for increased DHM delivery by co-crystal formulation. (a) DHM aggregates resist dissolution and persist in unstirred layer, thereby impeding intestinal absorption. (b) DHM-TEA crystals dissolve more readily in water, thereby increasing fraction of DHM available for absorption, which is beneficial to allow my DHM to reach the systemic circulation.

SUPPLEMENTARY TABLES

Supplementary Table 1. List of pH buffers.

**Buffer Preparation (in 80mL H_2O)**								
**pH**	**3.1**	**4.3**	**5.8**	**7.5**	**8.4**	**9.4**	**10.3**	**13.3**
Components and q.s.	0.48g Citric Acid<br>0.81g NaOH<br>0.51g HCl<br>Adjust to final pH, add water to 100mL	0.48g Citric Acid<br>0.81g NaOH<br>0.2g HCl<br>Adjust to final pH, add water to 100mL	0.48g Citric Acid<br>1.8g NaOH<br>0.46g HCl<br>Adjust to final pH, add water to 100mL	0.3g Tris-HCl<br>0.07g HCl<br>Adjust to final pH, add water to 100mL	0.3g Tris-HCl<br>0.03g HCl<br>Adjust to final pH, add water to 100mL	0.214g Na_2CO_3<br>1.17g $NaHCO_3$<br>Adjust to final pH, add water to 100mL	187.4mg Na_2CO_3<br>201.6mg $NaHCO_3$<br>Adjust to final pH, add water to 100mL	1.6g NaOH (0.05N NaOH)

Table 2. List of counterions and solvents used for counterion screen study.

Base/Counterion	Solvent
Triethanolamine (TEA)<br>pKa: 7.74<br>(MW: 149.188 g/mol)	Ethanol (A)
	Methanol (B)
	2-propanol (C)
	Acetone (D)
Sodium Hydroxide (NaOH)<br>pKa: 15.7<br>(MW: 39.99 g/mol)	Ethanol (A)
	Methanol (B)
	2-propanol (C)
	Acetone (D)
TRIS Base (2-Amino-2-(hydroxymethyl)-1,3-propanediol)<br>pKa: 8.1<br>(MW: 121.14 g/mol)	Ethanol (A)
	Methanol (B)
	2-propanol (C)
	Acetone (D)
L-Lysine<br>pKa: 10.53<br>(MW: 146.19 g/mol)	Ethanol (A)
	Methanol (B)
	2-propanol (C)
	Acetone (D)
Calcium Hydroxide (Ca $(OH)_2$)<br>pKa: 15.7<br>(MW: 74.09 g/mol)	Ethanol (A)
	Methanol (B)
	2-propanol (C)
	Acetone (D)

Table 3. MicroED data processing statistics of DHM-TEA.

Stoichiometric formula	C_{21} H_{27} N O_{11}
Mr	469.44
Temperature (K)	100
Crystal system, space group	Triclinic, P-1
Unit cell lengths a, b, c (Å)	a 8.0(10) b 11.1(10) c 12.2(10)
angles α, β, γ (°)	α 98.1(10) β 101.4(10) γ 106.4(10)
Cell volume (Å^3)	996.241
Overserved reflections (#)	10956
Unique reflections (#)	4415
R_{obs} (%)	14.5
R_{meas} (%)	18.5
CC1/2	98.9
Resolution (Å)	0.74
Completeness (%)	86.7
R_1 (%)	19.5
wR_2 (%)	46.6
GooF	1.562
